# The broad-spectrum rice blast resistance (*R*) gene *Pita2* encodes a novel R protein unique from *Pita*

**DOI:** 10.1186/s12284-020-00377-5

**Published:** 2020-03-13

**Authors:** Xiuli Meng, Gui Xiao, Mary Jeanie Telebanco-Yanoria, Paolo Miguel Siazon, Jonas Padilla, Rina Opulencia, Joseph Bigirimana, Georges Habarugira, Jun Wu, Mingyang Li, Baohua Wang, Guo-dong Lu, Bo Zhou

**Affiliations:** 1 State Key Laboratory of Ecological Pest Control for Fujian and Taiwan Crops, College of Plant Protection, Fujian Agriculture and Forestry University (FAFU), Fuzhou, 350002 China; 2 International Rice Research Institute (IRRI), DAPO Box 7777, 1301 Metro Manila, Philippines; 3 China National Hybrid Rice R&D Center/ Hunan Hybrid Rice Research Center, Changsha, 410125 China; 4 Institute of Biological Sciences, University of the Philippines Los Baños, College, Laguna, Philippines

**Keywords:** *Pita2*, Suppressor mutants, Rice blast, *R* gene diagnosis

## Abstract

**Background:**

Rice blast is generally considered the most devastating rice disease worldwide. The development of resistant varieties has been proven to be the most economical strategy to control the disease. A cluster of resistant (*R*) genes on rice chromosome 12 including *Pita, Pita2* and *Ptr* has been studies for decades. However, the relationship between these *R* genes has not been well established.

**Results:**

In this study, we compared the resistance spectra controlled by *Pita2* and *Pita* by testing their monogenic lines (MLs) in four hotspots found in the Philippines and Burundi from 2014 to 2018. The reaction patterns were distinct in two countries and that *Pita2*-mediated field resistance was relatively prevalent. Pathogenicity tests using 328 single-spore isolates in greenhouse further verified that IRBLta2-Re for *Pita2* conferred a relatively broader spectrum resistance than those of *Pita*. Rough and fine mapping of *Pita2* were conducted using F_2_ and F_3_ populations derived from IRBLta2-Re [CO] and CO 39 consisting of 4344 progeny to delimit *Pita2* in a genomic interval flanked by two markers 12 g18530 and 12 g18920 proximal to the centromere of chromosome 12. Alignment of the markers to the genomic sequence of IR64, which harbors *Pita2* verified by genetic analysis, approximately delimited the candidate gene(s) within 313-kb genomic fragment. The two *Pita2* suppressive mutants that contain mutations within *Pita2* were verified and identified. Comparative sequence analysis in these two mutants further identified that each individual allele contains a single nucleotide substitution at a different position resulting in nonsense and missense mutations in the protein product of LOC_Os12g18729. On the contrary, no sequence mutation was detected in other candidate genes, indicating that mutations in LOC_Os12g18729 were responsible for the loss of function of *Pita2*. *Pita2* encodes a novel R protein unique from *Pita*, which is exactly identical to the previously cloned *Ptr*. Moreover, based on the resistance gene analysis of rice varieties and mutants containing *Pita*, it was found that *Pita2* rather than *Pita* was responsible for the specificity to some differential isolates with *AvrPita*. The diagnosis and survey of *Pita2* in IRRI released varieties showed relatively low frequency, implying a high value of its application for breeding resistant varieties against rice blast via marker assisted selection.

**Conclusion:**

Our study clarified the relationship between *Pita*, *Pita2* and *Ptr*. *Pita2* is identical to *Ptr* and distinct from *Pita* in both sequence and chromosomal location although *Pita2* and *Pita* are genetically linked to each other. The loss of function of *Pita2* but not *Pita* eliminate the specificity to some *AvrPita* containing isolates, however, the mechanism underlying the recognition between *Pita2/Pita* and *AvrPita* remains elusive.

## Background

Plants have evolved two-branched of innate immune response to protect themselves against pathogens, including fungi, bacteria, viruses, and insects. The first branch recognizes pathogen-associated molecular pattern (PAMP) and triggers PAMP-triggered immunity (PTI), which generates the accumulation of reactive oxygen species (ROS) and the deposition of phenolic compounds (Jones and Dangl 2006). However, pathogens deliver effectors to suppress PTI (Bent and Mackey 2007). Resistant (*R*) genes in plant can recognize some of the effectors, referred to as avirulence (*Avr*) genes, and trigger the second branch of immune response called effector-triggered immunity (ETI), which can induce hypersensitive response (HR) to inhibit pathogen proliferation (Chisholm et al. 2006). Understanding the interaction between plants and pathogens can help to formulate better strategies to protect plants from diseases.

Rice blast caused by the fungal pathogen *Magnaporthe oryzae* destroys rice crops worldwide (Pennisi 2010). Incorporation of *R* genes into rice cultivars by marker assisted selection (MAS) presents an effective and economical way to control rice blast (Wang and Valent 2017). The bottleneck of this approach is that the *R* gene deployed in the varieties can be overcome in 2-4 years due to high level of variability in *Avr* genes of the pathogen (Skamnioti and Gurr 2009; Xiao et al. 2017). Therefore, continuous exploration of new *R* genes or alleles and utilization of effective ones in rice resistance breeding program are of great importance in controlling rice blast. As of now, more than 100 *R* genes have been mapped, 35 of which have been cloned (Wang et al. 2017). Except for *Pid2*, *pi21* and *Ptr*, all of cloned *R* genes encode proteins which contain nucleotide binding site-leucine-rich repeat (NBS-LRR) domain (Skamnioti and Gurr 2009; Liu et al. 2010; Wang et al. 2017). *Pi-d2* encodes a receptor-like kinase protein with a B-lectin and an intracellular serine-threonine kinase domain (Chen et al. 2006). *pi21* encodes a protein that contains a putative heavy metal-binding domain (Fukuoka et al. 2009) while *Ptr* encodes a protein with Armadillo repeats domain (Zhao et al. 2018).

Rice blast *R* genes tend to be clustered, specifically on chromosome 6, 11 and 12 in rice (Liu et al. 2010). The cluster of *R* genes linked to the centromere on chromosome 12 includes *Pita*, *Pita2* and at least eight other genes (Inukai et al. 1994; Yu et al. 1996; Li et al. 2008; Zheng et al. 2008; Koide et al. 2011; Lei et al. 2013; Dong et al. 2017). *Pita2* was first reported in the variety Pi No4 and mapped on the centromeric region on chromosome 12 (Kiyosawa 1967; Rybka et al. 1997). Some reports suggest that *Pita2* conferred a broader resistance spectrum than *Pita* (Kiyosawa 1967, 1971; Rybka et al. 1997)*. Pita2* was found resistant to all *Pita* avirulent isolates and some *Pita* virulent islolates (Kiyosawa 1967, 1971; Rybka et al. 1997). Furthermore, the resistance frequencies of *Pita2* and *Pita* differential varieties (DVs) to 119 isolates in the Philippines are 84% and 25.2%, respectively (Yanoria et al. 2008). The relationship between *Pita* and *Pita2* was reported to be closely linked or allelic (Rybka et al. 1997). Bryan et al. (2000) cloned *Pita* and proved that *Pita2* specificity is a combination of *Pita* and another linked *R* gene (Bryan et al. 2000). However, it is complicated to clone *Pita2* because all varieties containing *Pita2* such as Reiho, Katy and IR64, were found to contain *Pita* (Lee et al. 2009; Zhao et al. 2018)*.* The use of monogenic lines (MLs) and near-isogenic lines (NILs) of *Pita2* makes it easier to map and clone this gene. Tsunematsu et al. (2000) developed the first set of 29 MLs for 24 *R* genes. Among them, IRBLta2-Re and IRBLta-CT2 have been used in many studies for *Pita2* and *Pita* respectively (Lei et al. 2013; Mutiga et al. 2017). A series of NILs were developed under International Rice Research Institute (IRRI)-Japan Collaborative Research Project (Kobayashi et al. 2007; Yanoria et al. 2011). Of these NILs, IRBLta2-Re [CO] was one of the *Pita2* NILs at BC_6_F_21_ derived from Reiho and CO39 (Yanoria et al. 2011). On the other hand, 20 isolates that can differentiate various *R* genes in MLs were selected from 119 isolates of the Philippines (Yanoria et al. 2008). Among them, V86010 is avirulent to *Pita2* ML and can differentiate *Pita* and *Pita2* (Yanoria et al. 2008). *AvrPita* was cloned and proved to trigger resistance in *Pita2* rice as well as in *Pita* rice (Bryan et al. 2000; Orbach et al. 2000). Zhao et al. (2018) cloned *Ptr* which is required for *Pita* resistance and suggested that *Ptr* and *Pita2* are indistinguishable (Zhao et al. 2018). The mapping and cloning of *R* genes in *Pita* cluster has made significant progress. However, the precise relationship between *Pita, Pita2* and *Ptr* as well as the mechanism underlying the interaction between *AvrPita* and *Pita*, *Pita2* and *Ptr* require further investigation. In this study, we aimed to clarify the relationship between *Pita*, *Pita2* and *Ptr.* Cloning and uncovering the relationship of multiple genes in one cluster will be of great significance in elucidating the molecular interactions of these *R* genes, which will in turn guide the efforts of marker-assisted rice breeding for resistance.

## Results

### Distinct Reaction Patterns of *Pita2* against Rice Blast Populations in the Philippines and Burundi

To assess the resistance spectrum of *Pita2*, IRBLta2-Re was used to monitor its performance in four disease hotspots found in the Philippines (Bohol) and Burundi (other three sites, Table 1). IRBLta2-Re showed stable resistance in Bohol from 2016 to 2018 (Table 1) and in all sites in Burundi from 2014 to 2016. However, IRBLta2-Re became susceptible in all sites in Burundi in both 2017 and 2018, indicating that *Pita2* could have lost its effectiveness (Table 1). The distinct reaction patterns of *Pita2* against rice blast populations in the Philippines and Burundi could result from different dynamics of pathogen populations in these two countries. Compared to the field performance of *Pita2*, *Pita*, which was represented by two MLs (IRBLta-Zh and IRBLta-CT2), showed susceptibility in all sites during all years monitored, except in Bohol in 2016 (Table 1).

**Table 1:** Field resistance performance of *Pita2* against rice blast population in the Philippines and Burundi. R: resistant. S: susceptible. MR: moderate resistant. MS: moderate susceptible. –: no data

Year	Hotspots	Disease assessment to different IRBLs			
		IRBLta2-Re	IRBLta-Zh	IRBLta-CT2	LTH
2014	Cankuzo	R	S	–	S
	Gisha	R	S	–	S
	Rugombo	R	MS	–	S
	Bohol	–	–	–	–
2015	Cankuzo	R	S	MS	S
	Gisha	R	S	MS	S
	Rugombo	R	MS	S	S
	Bohol	–	–	–	–
2016	Cankuzo	MR	S	S	S
	Gisha	R	MS	S	S
	Rugombo	MR	S	S	S
	Bohol	R	–	R	S
2017	Cankuzo	S	S	MS	S
	Gisha	MS	MS	MS	S
	Rugombo	MS	MS	MS	S
	Bohol	MR	–	S	S
2018	Cankuzo	S	S	MS	S
	Gisha	MS	MS	MS	S
	Rugombo	S	S	S	S
	Bohol	R	–	MS	S

*R* resistant. *S* susceptible. *MR* moderate resistant. *MS* moderate susceptible. –: no data

Spraying inoculation study using single spore isolates showed that compared to other MLs, IRBLta2-Re showed relatively high resistance frequencies (RFs) ranging from 64% to 75% against 328 isolates collected in 2015 and 2017 (Table 2). In contrast, IRBLta-CT2 showed low RFs ranging from 32% to 35% (Table 2). The consistencies in the results obtained from the field studies with those from the greenhouse assessment using single spore isolates led us to conclude that the IRBLta2-Re controls a broader spectrum resistance than IRBLta-CT2 against rice blast isolates in the Philippines.

**Table 2:** Resistance frequency of different IRBLs to the rice blast isolates collected in Bohol in 2015 and 2017. -: no target *R* gene

IRBLs.	Target *R* genes	Resistance frequency (%)	
		2015(250 isolates)	2017(78 isolates)
IRBLta-CT2	*Pita*	35	32
IRBLta2-Re	*Pita2*	64	75
IRBLa-A	*Pia*	1	3
IRBLi-F5	*Pii*	99	94
IRBLks-S	*Piks*	4	3
IRBLk-ka	*Pik*	50	25
IRBLkp-K60	*Pikp*	33	25
IRBLkh-K3	*Pikh*	51	40
IRBLz5-CA	*Piz5*	86	92
IRBLzt-T	*Piz-t*	42	26
IRBLb-B	*Pib*	9	15
IRBLt-K59	*Pit*	9	20
IRBLsh-S	*Pish*	90	88
IRBL1-CL	*Pi1*	64	39
IRBL3-CP4	*Pi3*	98	90
IRBL5-M	*Pi5(t)*	96	98
IRBL7-M	*Pi7(t)*	43	34
IRBL9-W	*Pi9*	87	90
IRBL19-A	*Pi19*	37	18
IRBLkm-Ts	*Pikm*	50	30
IRBL20-IR24	*Pi20*	87	32
IRBL11-Zh	*Pi11*	5.3	10
LTH	*–*	0	0

### Genetic and Physical Delimitation of *Pita2* Proximal to the Centromere of Chromosome 12

To genetically map *Pita2*, an F_2_ population derived from the cross between IRBLta2-Re [CO] and CO39 was generated. Of the 2151 F_2_ progeny inoculated with the *Pita2*-avirulent isolate V86010, 1607 and 544 were resistant and susceptible, respectively. The segregation ratio of resistance and susceptibility was found to fit a 3:1 in the χ2 test of a goodness-of-fit (χ2 = 0.096, *P* = 0.75), indicating that the resistance of IRBLta2-Re [CO], i.e. *Pita2* against V86010, is controlled by a single locus/gene.

**Fig. 1:**
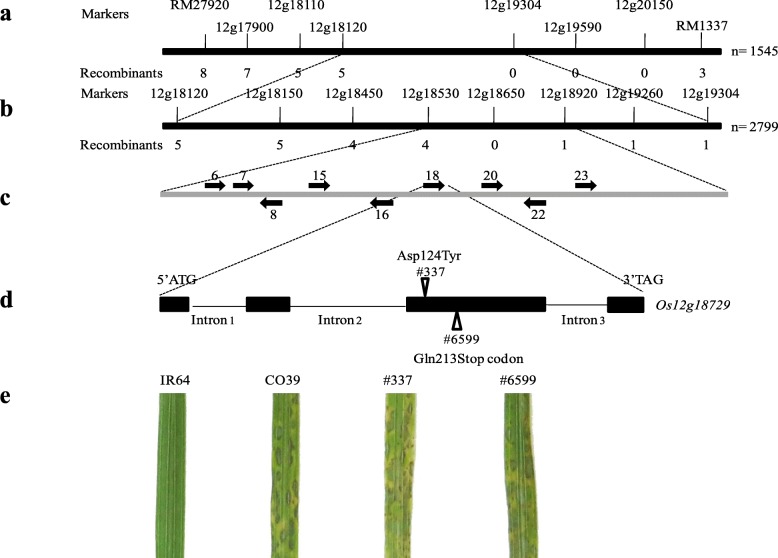
Mapping and identification of *Pita2.***a***Pita2* was mapped between 12 g18120 and RM1337 on chromosome 12. **b***Pita2* was fine mapped to 270 kb flanked by 12 g18530 and 12 g18920. The molecular markers and number of recombinants were listed above and underneath the thick line, respectively. The population size used for mapping is listed on the right. **c** Nine candidate genes of *Pita2* were identified in the genomic region of IR64 delimited by markers of 12 g18530 and 12 g18920. The transcriptional direction was indicated by arrows. **d** Diagram of *Pita2*. Two mutants of IR64 were indicated by triangle. The position of amino acid residue corresponding to the single nucleotide mutation was indicated. Exons were showed in filled boxes. **e** Reaction pattern of different varieties to 9475-1-3. The figures was not drawn in scale

Two steps of mapping were employed to genetically delimit the location of *Pita2*. In the first step, 544 susceptible and 1001 resistant progeny from the F_2_ population mentioned above were used for the genetic analysis. Eleven recombinants, including five resistant and six susceptible progenies were identified between two SSR markers of RM27920 and RM1337 (Additional file 1: Table S1). The phenotype of the F_3_ descendant population derived from five resistant F_2_ progeny all showed segregation in resistance and susceptibility (Additional file 1: Table S1). Based on the phenotype and genotype analyses, eight and three recombinants were assigned at RM27920 and RM1337, respectively, from *Pita2* (Fig. 1a and Additional file 1: Table S1). Six SNP markers between RM27920 and RM1337 were then developed and used for further screening of recombinants out of these 11 progeny, which helped to identify five and three recombinants at 12 g18120 and RM1337, respectively (Fig. 1a and Additional file 1: Table S1). No recombinants were identified at three markers within the genomic interval, indicating that *Pita2* was flanked by markers of 12 g18120 and RM1337 (Fig. 1a). In the second step, the derived segregating F_2_ progeny resistant to V86010 was advanced to F_3_. A total of 2799 lines were screened for the recombinants between two CAPS markers, 12g18120F3/R4- NheI and 12g19304F2/R2- ClaI. Six recombinants were identified between 12 g18120 and 12 g19304 (Additional file 2: Table S2). After phenotyping the F_4_ descendant population derived from each recombinant F_3_ progeny, five and one recombinants were identified at 12 g18120 and 12 g19034, respectively from *Pita2*, indicating that *Pita2* was delimited between these two markers (Fig. 1b and Additional file 2: Table S2). Six additional SNP markers were developed and used for the linkage analysis, which enabled the identification of four and one recombinants at 12 g18530 and 12 g18920, respectively, from *Pita2* (Fig. 1b and Additional file 2: Table S2). No recombinants between 12 g18650 and *Pita2* were identified, verifying the delimitation of *Pita2* in this genomic interval (Fig. 1b and Additional file 2: Table S2). By aligning the two flanking markers (12 g18530 and 12 g18920) along the genomic sequence of Nipponbare, the delimited *Pita2*-containing genome interval was determined to be approximately 270 kb in length (position: 10,712,341 to 10,982,783 bp, http://rice.plantbiology.msu.edu) on chromosome 12. It is worth noting that the centromere of chromosome 12 was previously found residing in the BAC clone of OsJNBa0088J04, which spanned the genome interval at the positions from 11,871,508 to 12,007, 390 bp and was approximately 1 Mb apart from the putative *Pita2* region.

**Fig. 2:**
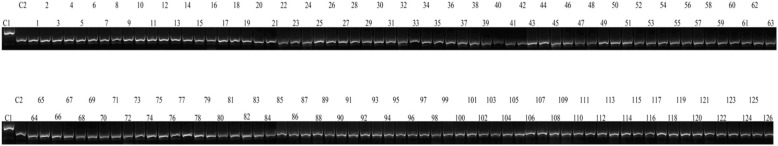
Linkage analysis of *R* gene in IR64 to 9475-1-3 with *Pita2* diagnosed CAPS marker (18729F3/R4- PvuII). F2 progeny derived from a cross between IR64 and CO39 were inoculated with 9475-1-3 and 126 susceptible lines were selected for linkage analysis. C1: IR64. C2: CO39. The CAPS marker has PvuII cleavage site on CO39 sequence. The product amplified by 18729F3/R4 from CO39 is 825 bp in size. After. PvuII cleavage, the products are 416 bp and 409 bp in size

An F_2_ population derived from the cross between IR64 and CO39 was generated to validate whether the location of *Pita2* in IR64 is located in the same genomic interval since IR64 was reported to harbor *Pita2* and its genomic sequence is available. A total of 722 F_2_ progeny were inoculated with the *Pita2*-avirulent isolate 9475-1-3 and separated into 561 and 161 resistant and susceptible progeny, respectively. The ratio of the number of resistant versus susceptible progeny fitted perfectly 3:1 (χ2 = 2.81, *P* = 0.094), indicating that, most likely, there is only a single pair of *R/Avr* involved in the resistance of IR64 against 9475-1-3. Moreover, a CAPS marker (18729F3/R4-PvuII), which was derived from the gene model 12 g18729 and located between markers of 12 g18530 and 12 g18920, was developed and used for the genetic analysis (Fig. 2). All the 126 susceptible F_2_ progeny showed CO39 pattern, suggesting that *Pita2* in IR64 was likely co-localized within the same genomic interval as identified in IRBLta2-Re [CO] (Fig. 2). The allelic region in IR64 was then retrieved from the IR64 genomic sequence (Schatz et al. 2014), which is approximately 313 kb in length, to further identify the candidate genes of *Pita2*.

### Identification of Candidate Genes of *Pita2* in IR64

We employed the program of Fgenesh (http://www.softberry.com) for gene prediction in the delimited region in IR64. A total of 43 gene models were identified, 17 of which corresponded to typical repetitive sequences (Additional file 3: Table S3). Moreover, other five gene models encoded small proteins with less than 100 amino acids in length. These two groups of gene models were not included in the further analysis. Except for two flanking genes, the sequences of the remaining 21 unique gene models were then compared to their alleles in the susceptible rice varieties, first with Nipponbare. Many of the 21 genes have their alleles with annotated gene models in Nipponbare. However, fours gene models, i.e., IR64–6, − 7, − 8, and − 16, were not predicted in Nipponbare. Further sequence analysis revealed that, except for IR64–6, the other three gene models were truly present in the genome sequence of Nipponbare albeit not predicted mainly due to frame shift sequence insertion/deletion. Sequence comparison between the gene models in IR64 and Nipponbare identified that the four gene models, IR64–28, − 34, − 36, − 38 were identical in protein sequence to LOC_Os12g18820, LOC_Os12g18860, LOC_Os12g18880, and LOC_Os12g18900 (http://rice.plantbiology.msu.edu/), respectively. Hence, these four gene models were not further considered as *Pita2* candidates. To further shortlist the candidate genes, we compared the remaining 11 gene models to the genome sequence of another susceptible variety CO39 derived from the second generation of sequence assembly (GenBank accession number: SWLY00000000), which enabled the identification of other three gene models, namely, IR64–10, − 11, and − 26, to their respective alleles in CO39. Moreover, the three gene models IR64–4, − 9, and − 17 did not have sequence polymorphisms unique to their respective alleles in both Nipponbare and CO39, suggesting that these sequence polymorphisms could not be associated with the functional differences. Taken together, two rounds of sequence comparisons shortlisted nine gene models as candidates of *Pita2* (Fig. 1c and Additional file 3: Table S3).

### Identification of LOC_Os12g18729 as *Pita2* by Characterization of Loss of Function Mutants Derived from EMS-Treated IR64 Mutant Population

Over 12,000 M_2_ individual plants were generated from EMS treated M_1_ plants of IR64 and screened to select susceptible mutants against 9475-1-3, the same isolate used for the genetic analysis of *Pita2* in IR64. Two M_2_ lines, namely, #337 and #6599 that showed, respectively, 29:2 and 35:4 segregation ratio of resistance versus susceptibility in M_2_ progeny were identified. The susceptible progeny were then recovered and used for further characterization. First, genetic validation of these two mutants was conducted to determine whether the mutation occurred in the *Pita2* gene itself or by its signaling components. These two mutants were crossed with CO39 to generate its respective F_1_ populations. Fifty-seven #337-derived and thirty-five #6599-derived F_1_ progeny were susceptible to 9475-1-3, indicating that CO39 could not complement the resistance in the F_1_ progeny. It can be speculated that the mutations in the *Pita2* gene itself rather than in the putative signaling components in #337 and #6599 led to the loss of resistance of IR64. Secondly, a set of IRBLta2-Re avirulent isolates were selected to verify their reactions to both mutants. As shown in Tables 3, 13 out of 22 isolates reverted to virulence when inoculated with both #337 and #6599, indicating that IR64 triggered resistance against these isolates through a single *R/Avr* pair, i.e., *Pita2*/*AvrPita2*. The disruption of *Pita2* resulted in the loss of function to those isolates. In contrast, additional *R/Avr* gene pairs to *Pita2*/*AvrPita2* existed in the interaction between IR64 and other nine isolates (IK81–25, M101–1–2-9-1, B90036, B90002, B90165, B90324, V850196, V86010 and C9240–4). Lastly, sequencing showed that the gene model IR64–18, i.e., LOC_Os12g18729, contained sequence mutations in different positions in #337 and #6599 compared to the one in the wild type IR64. The point mutation from guanine (G) to thymine (T) in LOC_Os12g18729 in #337 resulted in the amino acid change from aspartic acid (Asp) to tyrosine (Tyr) at 124th position. In #6599, the point mutation from cytosine (C) to thymine (T) resulted in the generation of a premature stop codon from glutamine (Gln) in LOC_Os12g18729 at the 213rd amino acid position (Fig. 1d and e). On the contrary, no sequence mutations were identified in the other eight candidate genes. Therefore, we postulated that the different mutations of LOC_Os12g18729 caused the loss of function of *Pita2* in #337 and #6599, and that LOC_Os12g18729 was most likely responsible for the function of *Pita2*.

**Table 3:** Pathogenicity test of differential isolates to different *Pita2* lines

Isolates	Different rice varieties					
	IR64	IRBLta2-Re	LTH	CO39	#337	#6599
9475-1-3	R	R	S	S	S	S
BN111	R	R	S	S	S	S
M64–1–3-9-1	R	R	S	S	S	S
B90019^a^	R	R	S	S	S	S
B90187	R	R	S	S	S	S
M39–1–3-8-1	R	R	S	S	S	S
JMB8401	R	R	S	S	S	S
JMB840610	R	R	S	S	S	S
M39–1–2-21–2	R	R	S	S	S	S
B90027^a^	R	R	S	S	S	S
92,319–9	R	R	S	S	S	S
B90033^a^	R	R	S	S	S	S
IK81–3	R	R	S	S	S	S
IK81–25^a^	R	R	S	S	R	R
M101–1–2-9-1	R	R	S	S	R	R
B90036	R	R	S	S	R	R
B90002	R	R	S	R	R	R
B90165	R	R	S	S	R	R
B90324	R	R	S	S	R	R
V850196	R	R	S	S	R	R
V86010	R	R	S	S	R	R
C9240–4	R	R	S	R	R	R

^a^These isolates contained *AvrPita* as indicated in Table 2. R: resistant. S: susceptible

### *Pita2* Encodes a Unique R Protein and Is Identical to *Ptr*

The predicted coding sequence of *Pita2* in IR64 is 2718 bp in length and composed of four exons (Fig. 1d), which were verified by sequencing the transcript obtained from the 3-week-old seedling (data not shown). The derived protein product contained 905 amino acid residues, and predicted to contain three strong transmembrane helices located at positions 85–106, 165–185, and 214–232 amino acids (https://embnet.vital-it.ch/cgi-bin/TMPRED_form_parser). However, the subcellular localization of *Pita2* was predicted inconsistently by different programs either in the cytoplasm (https://www.predictprotein.org/visual_results?req_id=686316ic) or extracellularly (http://www.softberry.com/cgi-bin/programs/proloc/protcomppl.pl). It is worth noting that *Pita2* was identical to *Ptr* as described previously, which was found to contain an Armadillo (ARM) repeat domain (Zhao et al. 2018). No significant sequence similarity was found between *Pita* and *Pita2*. The expression of *Pita2* in IRBL28, CN16, and IR64 was investigated and found that *Pita2* was expressed constitutively at different time points after infection of rice blast (Additional file 4: Fig. S1). The constitutive expression of *Pita2* was consistent with the finding described previously (Zhao et al. 2018).

### Analysis of *Pita2* Haplotypes in IRBLs Containing *Pi19* or its Tentative Alleles

A comprehensive analysis of haplotype diversity of *Ptr* was conducted previously (Zhao et al. 2018). In this study, we investigated the sequence variation of *Pita2* haplotypes in 4 monogenic lines (IRBLkp-K60, IRBLzt-T, IRBLta-K1, IRBL19-A) which were characterized to each contain *Pi19* or its functional allele previously (Selisana et al., 2017) and two susceptible rice varieties (LTH and CO39). The *Pita2* haplotypes in all these 6 rice varieties were found to belong to the haplotype group containing a 12-bp insertion (Table 4), which was characterized to be associated with the pathogen recognition specificity as described previously (Zhao et al. 2018). In addition to this 12-bp insertion, they contained a varied number of residues different from *Pita2* in Katy or IR64 (Table 4). Sequence comparison further revealed that some rice varieties contained identical* Pita2* haplotypes. For example, Nipponbare and IRBLzt-T contained an identical* Pita2 *haplotype whereas IRBL19-A and IRBLkp-K60 contained another identical *Pita2* haplotype (Table 4 and Additional file 5: Fig. S2). It is worth noting that the *Pita2* haplotype in CO39, which was more closely related to* Pita2 *in IR64 or Katy (Additional file 5: Fig. S2), was found to be responsible for the resistance of CO39 to some avirulent isolates based on the functional analysis of CRISPR line (Gui et al., unpublished), implying that the *Pita2* haplotype in CO39 is a novel functional allele of *Pita2*. It is elusive whether other *Pita2* haplotypes identified in four monogenic lines containing *Pi19* or its alleles are responsible for the resistance of their respective varieties.

**Table 4:** Amino acid polymorphism of Pita2 in different rice germplasm

Varieties	Amino acid position																									
	789	792	793	846	847	851	854	858	866	869	870	871	872	873	875	877	879	881	882	886	887	889	891	895	897	899
Katy	E	L	R	T	T	M	A	N	I	M	–	–	–	–	E	P	K	E	P	M	P	K	P	Y	F	V
IR64	**.**	**.**	**.**	**.**	**.**	**.**	**.**	**.**	**.**	**.**	–	–	–	–	**.**	**.**	**.**	**.**	**.**	**.**	**.**	**.**	**.**	**.**	**.**	**.**
CO39	**.**	**.**	**.**	**.**	**.**	**.**	**.**	**.**	**.**	K	K	P	E	K	K	A	R	**.**	L	**.**	**.**	**.**	**.**	**.**	**.**	**.**
Nipponbare	**.**	**.**	**.**	**.**	**.**	**.**	**.**	**.**	**.**	K	K	P	E	K	K	A	R	**.**	L	V	T	**.**	T	F	**.**	G
LTH	K	Q	G	A	A	V	T	K	M	K	K	P	E	E	K	A	S	A	L	**.**	**.**	Y	**.**	F	Y	S
IRBLkp-K60	**.**	**.**	**.**	**.**	**.**	**.**	**.**	**.**	**.**	K	K	P	E	K	K	A	R	**.**	L	V	**.**	**.**	T	F	**.**	G
IRBLzt-T	**.**	**.**	**.**	**.**	**.**	**.**	**.**	**.**	**.**	K	K	P	E	K	K	A	R	**.**	L	V	T	**.**	T	F	**.**	G
IRBLta-K1	**.**	**.**	**.**	**.**	**.**	**.**	**.**	**.**	**.**	K	K	P	E	K	K	A	R	**.**	L	V	T	**.**	**.**	**.**	**.**	G
IRBL19-A	**.**	**.**	**.**	**.**	**.**	**.**	**.**	**.**	**.**	K	K	P	E	K	K	A	R	**.**	L	V	**.**	**.**	T	F	**.**	G

*LTH*: Lijiangxintuanheigu; *IRBL*: IRRI bred blast resistant line. “-” denotes deletion and “.” denotes identical to the one of Ptr in Katy (Genbank accession no.: MG385185). Pita2 haplotypes in different rice varieties were cloned and sequenced in this stud except Nipponbare containing LOC_Os12g18729

### *Pita2* rather than *Pita* Conferred Resistance to some *AvrPita*-Containing Isolates

**Fig. 3:**
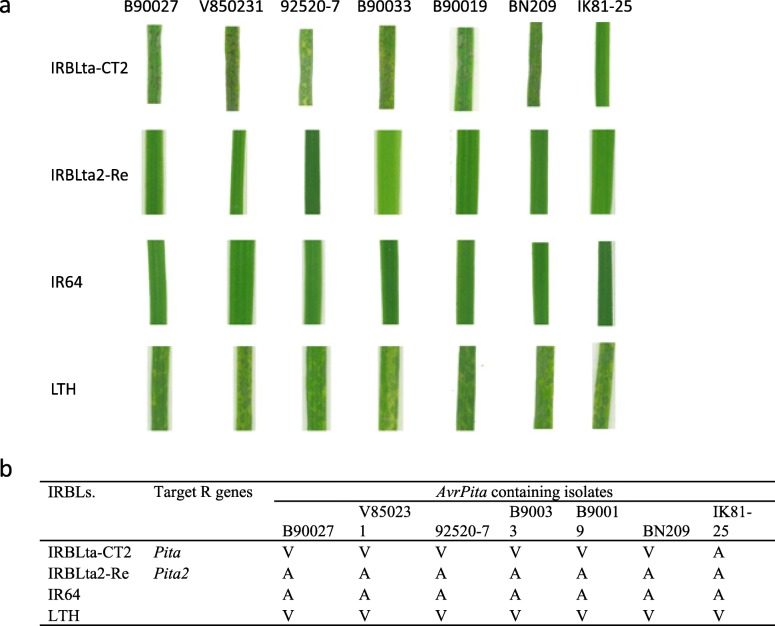
Disease reaction of *AvrPita* containing isolates to different rice lines. **a** Photograph showing disease reaction of 7 *AvrPita* containing isolates to different rice lines. **b** Disease reaction of 7 *AvrPita* containing isolates to different rice lines. *A* avirulent. *V* virulent

The identification of *Pita2* revealed that *Pita2* (corresponding to LOC_Os12g18729) and *Pita* (corresponding to LOC_Os12g18360) were encoded by different genes and separated from each other by approximately 210 kb in the genome sequence of Nipponbare. The recombinant 8-2B3, which was heterozygous at the *Pita2* locus but homozygous with the susceptible allele at the *Pita* locus, verified that the function of *Pita2* was independent from *Pita* (Additional file 2: Table S2). To clarify the contribution of resistance spectrum conferred by *Pita* and *Pita2*, two MLs and one cultivar (IRBLta-CT2, IRBLta2-Re, IR64) were used for resistance spectrum analysis. The presence of *Pita* and *Pita2* were verified by PCR amplification and subsequent amplicon sequencing, which revealed that both IR64 and IRBLta2-Re contained both *Pita* and *Pita2* as described previously (Lee et al. 2009; Zhao et al. 2018). However, IRBLta-CT2 contained only *Pita* as reported previously (Lee et al. 2009). A single T base-pair deletion corresponding to the position of 554 in the coding sequence of *Pita2* in IR64 was identified in IRBLta-CT2, leading to a frame shift in the coding sequence and a disrupted allele of *Pita2*. A set of seven isolates containing *AvrPita* was used for pathogenicity test using these rice varieties and the susceptible check variety, LTH (Fig. 3). Table 3 shows that both IR64 and IRBLta2-Re were resistant to all isolates. Intriguingly, IRBLta-CT2 was susceptible to all isolates except IK81–25 (Fig. 3), suggesting that *Pita* in IRBLta-CT2 did not function as it did in IRBLta2-Re and IR64. It is reasonable to assume that additional *R* gene(s) could contribute to the resistance of IRBLta-CT2 to IK81–25. Moreover, it is worth noting that two *Pita2* disrupted mutants (#337 and #6599) were susceptible to four of these *AvrPita* isolates, except IK81–25 (other three isolates were not included in the analysis), indicating that disruption of *Pita2* resulted in the loss of resistance to those isolates (Table 3). However, it is still elusive whether this change from resistance to susceptibility of the mutants to the *AvrPita* isolates is due to the recognition between *Pita2* and *AvrPita* or another unknown avirulence effector.

### The Frequency of *Pita2* in Modern IRRI Released Varieties

To investigate the frequency of *Pita2* in improved varieties, we used the InDel marker for *Ptr*, Z12F/R (Zhao et al. 2018) in the diagnosis of a set of 46 IRRI varieties. There were 14 out of 46 selected IRRI varieties resolved a PCR amplicon similar to that of IR64 (Table 5 and Additional file 6: Fig. S3). On the contrary, 32 varieties resolved an amplicon similar to that of CO39, which was 12 bp larger than that of IR64 by referring to their sequence differences of alleles of *Pita2*. A set of seven differential isolates, including five avirulent and two virulent isolates to *Pita2,* was used to evaluate the resistance spectrum of these 46 varieties. The rice lines that resolved a smaller amplicon were resistant to all five *Pita2*-avirulent isolates (Table 5 and Additional file 6: Fig. S3). However, at least one of the five isolates was virulent to the rice lines resolving a larger amplicon. We speculate that the haplotype of *Pita2* present in the 34 rice varieties that resolved a larger amplicon did not confer the function of *Pita2* as identified in IR64. In this regard, the frequency of *Pita2* in IRRI released varieties was approximately 30%. The sequence corresponding to the 4th exon of *Pita2* where the InDel resided was further amplified using the primer pair 18729F3/R4 and subsequently determined. The result identified three different haplotypes in the 34 rice varieties. The first haplotype was found in two varieties (NSIC Rc 220SR and NSIC Rc 304SR) identical to that of CO39. The second haplotype was found in six varieties (NSICRc300, NSICRc340, NSICRc192, NSICRc280, NSICRc288 and NSICRc342) that contained a fragment deletion outside the InDel. The last one was found in 26 rice varieties that contained a single nucleotide change (from “G” to “C”) when compared to that of CO39. Intriguingly, this nucleotide change depleted the cleavage site of PvuII. Thus, CAPS marker (18729F3/R4-PvuII) was developed to differentiate these haplotypes (Table 5 and Additional file 6: Fig. S3) and can be used for the introgression of *Pita2* via marker assisted selection.

**Table 5:** Genotype and phenotype of IRRI new released varieties

Entry No	Variety	Genotype using InDel and CAPS markers		Phenotype to differential isolates						
		Z12F/R	18729F3/R4-PvuII	PO6–6	CA89	BN209	IK81–25	M39–1–3-8-1	M101–1–2-9-1	BN111
	IR64	D	N	S	S	R	R	R	R	R
	CO39	I	Y	S	S	S	S	S	S	S
	IRBLta2-Re [CO]	D	N	S	S	R	R	R	R	R
PR 1	NSIC Rc 216	I	N	S	R	R	R	R	R	S
PR 2	NSIC Rc 222	I	N	S	R	R	R	R	R	S
PR 3	NSIC Rc 224	D	N	R	R	R	R	R	R	R
PR 4	NSIC Rc 226	I	N	S	R	R	R	R	R	S
PR 5	NSIC Rc 238	D	N	R	R	R	R	R	R	R
PR 6	NSIC Rc 240	D	N	S	R	R	R	R	R	R
PR 7	NSIC Rc 298	I	N	S	S	R	R	R	R	S
PR 8	NSIC Rc 300	I	d	S	S	R	R	S	S	S
PR 9	NSIC Rc 302	I	N	S	S	S	R	R	S	R
PR 10	NSIC Rc 308	I	N	S	S	R	R	R	R	S
PR 11	NSIC Rc 352	I	N	S	S	R	R	R	R	S
PR 12	NSIC Rc 356	D	N	R	R	R	R	R	R	R
PR 13	NSIC Rc 182	I	N	S	R	R	R	R	R	S
PR 14	NSIC Rc 184	I	N	S	S	S	R	R	S	S
PR 15	NSIC Rc 290	D	N	S	S	R	R	R	R	R
PR 16	NSIC Rc 294	I	N	S	S	R	R	R	R	S
PR 17	NSIC Rc 296	I	N	S	S	R	R	R	R	S
PR 18	NSIC Rc 324	I	N	S	S	R	R	R	R	S
PR 19	NSIC Rc 328	D	N	R	R	R	R	R	R	R
PR 20	NSIC Rc 330	I	N	S	R	R	R	R	R	R
PR 21	NSIC Rc 332	D	N	S	R	R	R	R	R	R
PR 22	NSIC Rc 334	I	N	R	S	R	R	R	R	S
PR 23	NSIC Rc 336	I	N	S	S	R	R	R	R	R
PR 24	NSIC Rc 338	D	N	S	S	R	R	R	R	R
PR 25	NSIC Rc 340	I	d	S	S	R	R	R	R	R
PR 26	NSIC Rc 392	D	N	S	S	R	R	R	R	R
PR 27	NSIC Rc 192	I	d	R	S	S	R	S	S	R
PR 28	NSIC Rc 272	I	N	R	S	R	R	R	R	R
PR 29	NSIC Rc 274	I	N	R	R	R	R	R	R	R
PR 30	NSIC Rc 278	D	N	S	S	R	R	R	R	R
PR 31	NSIC Rc 280	I	d	S	S	R	R	R	S	S
PR 32	NSIC Rc 282	D	N	S	S	R	R	R	R	R
PR 33	NSIC Rc 284	I	N	S	S	R	R	R	R	S
PR 34	NSIC Rc 286	I	N	S	S	R	R	R	R	S
PR 35	NSIC Rc 288	I	d	S	S	R	S	S	S	R
PR 36	NSIC Rc 238	D	N	R	R	R	R	R	R	R
PR 37	NSIC Rc 346	I	N	S	S	R	R	S	R	R
PR 38	NSIC Rc 25	I	N	R	R	R	R	S	R	R
PR 39	PSB Rc 18-Sub1	D	N	S	S	R	R	R	R	R
PR 40	NSIC Rc 21SR	I	N	S	S	R	R	R	R	S
PR 41	NSIC Rc 218SR	I	N	S	R	R	R	R	S	S
PR 42	NSIC Rc 220SR	I	Y	R	R	R	R	R	R	R
PR 43	NSIC Rc 304SR	I	Y	R	R	R	S	R	S	R
PR 44	NSIC Rc 342SR	I	d	S	S	R	R	S	S	R
PR 45	NSIC Rc 344SR	D	N	S	S	R	R	R	R	R
PR 46	Mestiso 30	I	N	R	S	R	R	R	R	S

I: Amplicon size is 226 bp; D: Amplicon size is 214 bp; N: no cleavage site with PvuII and DNA fragment size is 812 bp; Y: containing cleavage site of PvuII and fragments’ sizes are 416 bp and 408 bp; d: containing a small deletion outside InDel region; R: resistant; S: susceptible; PO6–6 and CA89 are virulent isolates to Pita2 whereas other 5 isolates are avirulent

## Discussion

### *Pita2* encodes a Sequence and Genomic Location Unrelated R Protein of *Pita*

*Pita2* was firstly reported in the variety Pi No4, which was allelic to *Pita* in another variety, Pi No1, and mapped to the centromeric region on chromosome 12 (Kiyosawa 1967; Rybka et al. 1997). Several other *R* genes such as *Pi-4a(t)*, *Pi-4b(t)*, *Pi-6(t)*, and *Pi-12(t)*, *Pi57(t)*, *Pi19(t)*, *Pi61(t)*, *Pi20(t)* were also identified to be allelic or linked to *Pita* and *Pita2* (Inukai et al. 1994; Yu et al. 1996; Li et al. 2008; Zheng et al. 2008; Koide et al. 2011; Lei et al. 2013; Dong et al. 2017). The cloning of *Pita* revealed that it encoded an NBS-LRR type R protein, which made a significant progress in the dissection of this genetic complex harboring multiple *R* genes (Bryan et al. 2000). Zhao et al. (2018) recently reported the cloning of a new broad-spectrum *R* gene *Ptr*, which encoded a novel R protein containing an ARM repeat domain. *Ptr* was found to be required for the function of *Pita* and its disruption depleted the function to *AvrPita-*containing strains (Zhao et al. 2018). However, the relationship between *Pita* and *Pita2* as well as other *R* genes remains elusive. In this study, we mainly utilized genetic approach to differentiate the *Pita2* gene in the *Pita2* containing varieties, IRBLta2-Re [CO], IR64 and IRBLta2-Re. The use of a large genetic population enabled the delimitation of *Pita2* into a small genomic interval. More importantly, the recombinants between *Pita2* and *Pita* which are identified in the population broke the association of *Pita2* from *Pita*, allowing the clarification of the relationship between them. For example, the identification of the recombinant 8-2B3 verified that *Pita2* was independent from *Pita* (Additional file 2: Table S2). Moreover, the generation and characterization of two *Pita2* independent suppressor mutants provided convincing evidence that the single gene/locus conferring resistance in IR64 against the isolates was controlled by *Pita2*. The set of isolates avirulent to the IRBLta2-Re which was often used for the diagnosis of the effectiveness of *Pita2* in the field and green house gained virulence to two *Pita2* suppressor mutants, further verifying that resistance of IR64 against these diagnostic isolates was attributable to *Pita2* rather than on other *R* genes in IR64 (Table 3). The molecular characterization of *Pita2* confirmed that it was distinct from *Pita* based on the sequence and genomic location, although they were tightly linked in the approximately 200 kb genomic interval, clarifying the genetic relationship between *Pita* and *Pita2*. The allelism test among *Pi19* in IRBL19-A, *Pi20* in IRBL20-IR24, and *Pita2* in IRBLta2-Re revealed that *Pi19*, *Pi20*, and *Pita2* were most likely allelic to each other (unpublished), implicating that *Pi19* and *Pi20* could be alleles of *Pita2*. It was found that *Ptr* being verified as *Pita2* in this study contained abundant variations in diverse rice germplasm (Zhao et al. 2018). The molecular characterization of *Pita2* paved a way in functional characterization of these haplotypes using sequence-based approaches, e.g., CRISPR and gene complementation tests.

### How *Pita2* and *Pita* Contribute to the Specificity to Isolates Containing *AvrPita* Is Still Elusive

Based on pathogenicity tests in both field and greenhouse, it is demonstrated that IRBLta2-Re showed higher resistance frequency than IRBLta-CT2, indicating that *Pita2* conferred a broader spectrum resistance than *Pita*. Previous studies indicated that *Pita* resistance was also found in *Pita2* varieties, suggesting *Pita2* resistance specificity could be a combination of *Pita* and at least another *R* gene (Bryan et al. 2000). Intriguingly, the loss of function of *Ptr* compromised the resistance of both *Pita* and *Pita2*, suggesting that *Ptr* was required for the function of *Pita* (Zhao et al. 2018). In this study, it is demonstrated that the isolates with *AvrPita* gained virulence to *Pita2* suppressor IR64 mutants and IRBLta-CT2 in which *Pita2* was truncated due to a single nucleotide deletion in the coding region (Table 3 and Fig. 3), indicating that *Pita2* was required for the function of *Pita*. The verification of *Ptr* being *Pita2* explained the findings of this study. In this regard, the resistance observed in IRBLta-CT2 could be not attributable to *Pita*, which may result in the complexity in the comparison of resistance spectrum between *Pita* and *Pita2*. However, it is doubtless that *Pita2* can also recognize non-*AvrPita* avirulence effectors in rice blast pathogen. For example, *AvrPita* was not identified in both V86010 and 9475-1-3 that were used in the genetic analysis of *Pita2* in this study. The hypothesis that *Pita2* is required for the function of *Pita* needs to be further verified. The knockout of *Pita* in rice varieties containing both *Pita* and *Pita2* can be helpful to finish the remaining pieces in this puzzle. It is also important to use transformed isolates with and without *AvrPita* to investigate their pathogenicity to rice lines with and without *Pita2* and *Pita*.

### Molecular Cloning of Different *R* Genes Clustered in the Same Genomic Complex Is Fundamentally Important for Precise Identification and Utilization in Resistance Breeding Program

Over 100 rice blast *R* genes were genetic mapped and designated in different rice varieties (Zhao et al. 2018). Due to the complexity of *R* genes and their clusters, less than 30 *R* genes were characterized at the molecular level (Su et al. 2015; Zhao et al. 2018). Due to the use of different set of isolates usually collected locally, the comparison of resistance spectrum conferred by different *R* genes becomes an impossible task. Thus, new *R* genes identified in unique donors are often designated to a new name using either a number following the latest one or the name of the origin as the suffix after the abbreviation of *Pi*. Moreover, many *R* genes with different designation reported by different groups were actually identical to each other. For example, in our previous study, we found that the broad-spectrum resistance observed in JaoHom Nin, one of the widely used donor for blast resistance, was controlled by two known *R* genes, *Pi7* and *Pish* (Chaipanya et al. 2017). It was also found that *Pigm* was actually identical in sequence to *Pi50* cloned earlier (Su et al. 2015; Deng et al. 2017). A comprehensive screening of *Pi2/9* alleles identified at least four rice varieties contained *Pi2-A15* identical to *Pi50/Pigm* based on sequence comparison (Xiao et al. 2017). It is therefore reasonable to assume that new *R* genes with different designation could be allelic or even identical to the previously characterized *R* genes. Thus, it is necessary to have a more standard nomenclature system to avoid the confusion particularly to rice breeders in selecting the correct genes in their breeding program. The sequence verification of known *R* genes linked to the newly identified *R* genes must be a prerequisite before a new gene designation is assigned.

In this study, the cloning of *Pita2* clarified its relationship to *Pita* and *Ptr*. Comprehensive sequence analysis of their haplotypes in the donor varieties containing *Pita*/*Pita2* linked *R* genes previously reported by different groups has effectively clarified the relationship of these *R* genes with *Pita* and *Pita2*. In this study, we identified three *Pita2* haplotypes in 46 IRRI varieties containing sequence differences from *Pita2*. The varieties containing these *Pita2* haplotypes were susceptible to *Pita2*-avirulent isolates. Sequences of these haplotypes were referred in the development of InDel and CAPS markers that can be used for the introgression of *Pita2* in these susceptible rice varieties.

## Conclusion

In this study, *Pita* and *Pita2* resistance spectra were assessed in the field and in the greenhouse. *Pita2* conferred a broader resistance spectrum than *Pita*. *Pita*2 was mapped approximately 313 kb genomic interval flanked by 12 g18530 and 12 g18920 on the short arm of chromosome 12 based on the equivalent sequence of IR64. LOC_Os12g18729 was verified as a single candidate of *Pita2* by characterizing suppressive mutants. The IRRI newly released varieties were assessed by *Pita2* diagnostic markers, which implicate that *Pita2* has a high value in future rice breeding. A novel *Pita2* allele was found in many IRRI varieties that have a single nucleotide change (from “G” to “C”) compared to the allele in CO39. A CAPS marker (18729F3/R4-PvuII) was developed that can distinguish different *Pita2* haplotypes. Taken together, our study clarifies the relationship between *Pita*, *Pita2* and *Ptr*. Moreover, the developed markers will be useful for the improvement of future blast resistance rice breeding programs.

## Methods and Materials

### Plant Materials and Isolates

Plant materials used in this study were listed in Additional file 7: Table S4. Twenty-three IRBLs harboring different resistant *R* genes in the background of LTH (Tsunematsu et al. 2000; Yanoria et al. 2010) and IRBLta2-Re [CO] harboring *Pita2* in the background of CO39 (Yanoria et al. 2011) were maintained at IRRI (https://www.irri.org). IR64 and 46 IRRI new released varieties were obtained from International rice genebank collection (IRGC) of IRRI. Three hundred and fifty-five *M. oryzae* isolates were collected in the different years and maintained at rice blast isolate collection at IRRI (Additional file 8: Table S5).

### Disease Evaluation in the Greenhouse and Field

The disease evaluation in the greenhouse followed Yanoria et al. (2008). Briefly, isolates were cultured in prune agar medium (three pieces prunes, 5 g alpha-lactose monohydrate, 1 g yeast extract, 21 g gulaman bar and 1 L distilled H_2_O) and spores were harvested using 0.02% (vol/vol) Tween 20 in distilled water. The final spore concentration for inoculation is 1 × 10^5^ conidia/ml. Rice seedlings at the three-to four-leaf stage were used for blast inoculation. Disease assessment was done 6-7 days after inoculation following the method of Bonman (1986) wherein 0 = no evidence of infection; 1 = brown specks smaller than 0.5 mm in diameter, no sporulation; 2 = brown specks about 0.5-1 mm in diameter, no sporulation; 3 = roundish to elliptical lesions about 1–3 mm in diameter with gray center surrounded by brown margins, lesions capable of sporulation; 4 = typical spindle-shaped blast lesions capable of sporulation, 3 mm or longer with necrotic gray centers and water-soaked or reddish brown margins, little or no coalescence of lesions; 5 = lesions as in 4 but about half of one or two leaf blades killed by coalescence of lesions. Scores of 0–3 were considered resistant reactions, and scores of 4 and 5 were considered susceptible reactions.

For field evaluation, four hotspots were chosen for monitoring: Bohol, in the Philippines and Gisha, Cankuzo and Rugombo, in Burundi. Three replications were set for every hotspot wherein each replicate containing 50 lines. Disease evaluation was done 40 days after transplanting based on the Standard Evaluation System (SES) IRRI (2013): 0–1: resistant; 3: moderately resistant; 5: moderately susceptible; 7: susceptible; 9: very susceptible.

### Designing Markers

Two SSR markers (RM27920 and RM1337) which were reported close to the centromere of chromosome 12 (Jia et al. 2012) and had polymorphism between IRBLta2-Re [CO] and CO39 were selected for rough mapping. For fine mapping *Pita2*, SNP and CAPS markers within delimited interval were designed. To make sure that the designed markers are specific on chromosomes, annotated expressed proteins in “rice functional genomic express database” (http://signal.salk.edu/cgi-bin/RiceGE5) were selected. Allelic region of these selected genes in IR64 and CO39 were compared and markers were designed based on the polymorphism between these two genomes. The SNP markers 12 g18120 and 12 g19304 were further developed into CAPS markers to screen more recombinants (Additional file 9: Table S6).

### Sequence Analysis

The polymorphic sequences in recombinants were amplified by PCR using SNP markers (Additional file 9: Table S6). Coding sequence of nine *Pita2* candidate genes in IR64, #337 and #6599 were amplified by PCR using primers (Additional file 9: Table S6). Sequencing was conducted by Biosune (http://www.biosune.com/) in China. BLASTN search program (https://blast.ncbi.nlm.nih.gov/Blast) and “sequencher 5.4.6” software (http://www.genecodes.com) were used for sequence alignment.

### EMS Mutagenesis and Mutants Screening

Approximately 12,000 seeds of IR64 were pre-soaked in an opaque bucket containing four liters of distilled water. This was incubated at 28 °C for 24 h. After incubation, the water was drained and replaced with a freshly prepared 4-l 0.8% EMS solution. The bucket was gently mixed to ensure that the solution would deeply penetrate the seeds and was incubated for 16 h. The mutagen solution was drained after incubation and the treated seeds were washed three times with distilled water. The solution and all the washings were collected for inactivation. Treated seeds were sown in pre-fertilized soil and allowed to grow for 21 days. The seedlings were transplanted in the field and the normal breeder’s practice was implemented for the field maintenance. The seedlings were allowed to grow until maturity. Seeds from surviving plant were collected as M2 population of each mutant line. Segregation analysis was done on M2 of each mutant line using isolate 9475-1-3.

### A High Throughput Protocol for Plant Genomic DNA Preparation and PCR

Rice seeds were pre-soaked for 48 h and sown in a 96-well PCR plate with cut bottom. Then, the PCR plate was kept in a plastic tray (60 cm × 38 cm × 12 cm) and plants were grown hydroponic solution (Shouichi et al. 1976). After 1 week, fresh leaves were cut into small pieces (1 mm wide) and place in another 96-well PCR plate with 40ul TPS buffer (100 mM Tris-HCl (PH8.0) 100 ml, 10 mM EDTA 20 ml, 1 M KCl 74.55 g, add water up to 1 L and sterilize it by autoclave). The mixture in PCR plate was then incubated at 95 °C for 5 min to extract DNA. LA Taq DNA polymerase (http://www.takara-bio.com/) was used following this system (10 x LA taq buffer 1ul, dNTP 1ul, DNA mixture 1.5ul, LA taq enzyme 0.1ul, forward primer 0.25ul, reverse primer 0.25ul, ddH_2_O 5.9ul). PCR was done following this PCR profile (95° 2 min, 98° 10s, 55° 30s, 68° 30s, 72° 10 min, 33 cycles).

## Supplementary information

**Additional file 1: Table S1.** Genotype and phenotype of 11 recombinants in the first round of genetic analysis

**Additional file 2: Table S2.** Genotype and phenotype of 6 recombinants in the second round of genetic analysis

**Additional file 3: Table S3.** Candidate genes of *Pita2*

**Additional file 4: Fig. S1.** Expression pattern of *Pita2* haplotypes in different rice varieties.

**Additional file 5: Fig. S2.** Phylogenetic tree analysis of the Pita2 haplotypes in different rice varieties.

**Additional file 6: Fig. S3.** Forty-six IRRI new released varieties were diagnosed by two *Pita*2 markers.

**Additional file 7: Table S4.** Plant materials

**Additional file 8: Table S5.** Isolates

**Additional file 9: Table S6.** Markers

## Data Availability

Not applicable.

## Abbreviations

ARM: Armadillo
Avr: Avirulence
DV: Differential Varieties
ETI: Effector Triggered Immunity
HR: Hypersensitive Response
LRR: Leucine-Rich Repeat
MAS: Marker Assisted Selection
ML: Monogenic Line
NBS: Nucleotide Binding Site
NIL: Near Isogenic Line
PAMP: Pathogen Associated Molecular Pattern
PCR: Polymerase Chain Reaction
PTI: PAMP -Triggered Immunity
R gene: Resistance gene
ROS: Reactive Oxygen Species
