# Supplementary material for: The broad-spectrum rice blast resistance (R) gene Pita2 encodes a novel R protein unique from Pita
Source: Rice (N Y). 2020 Mar 13;13:19. doi: 10.1186/s12284-020-00377-5 (PMC7070119; doi:10.1186/s12284-020-00377-5)
Supplement: Supplementary file 1 — Additional file 1: Table S1. Genotype and phenotype of 11 recombinants in the first round of genetic analysis [file 12284_2020_377_MOESM1_ESM.docx]

| **Table S1** Genotype and phenotype of 11 recombinants in the first round of genetic analysis. ^a^The phenotypes of F_3_ descendant population derived from resistant F2 progeny were assessed for determining the recombinants at different markers. R/S: segregation in resistance (R) and susceptibility (S). C: CO39 pattern. P: IRBLta2-Re[CO] pattern. | | | | | | | | | |
| --- | --- | --- | --- | --- | --- | --- | --- | --- | --- |
| Recombinants | Phenotype^a^ | Genotype pattern resolved by different markers | | | | | | | |
|  |  | RM27920 | 12g17900 | 12g18110 | 12g18120 | 12g19304 | 12g19590 | 12g20150 | RM1337 |
| R222 | R/S | C/C | C/C | C/P | C/P | C/P | C/P | C/P | C/P |
| R312 | R/S | C/C | C/C | C/C | C/C | C/P | C/P | C/P | C/P |
| R342 | R/S | C/C | C/C | C/C | C/C | C/P | C/P | C/P | C/P |
| R452 | R/S | C/C | C/C | C/C | C/C | C/P | C/P | C/P | C/P |
| R648 | R/S | C/C | C/C | C/P | C/P | C/P | C/P | C/P | C/P |
| S131 | S | C/P | C/C | C/C | C/C | C/C | C/C | C/C | C/C |
| S207 | S | C/C | C/C | C/C | C/C | C/C | C/C | C/C | C/P |
| S226 | S | C/C | C/C | C/C | C/C | C/C | C/C | C/C | C/P |
| S230 | S | C/C | C/C | C/C | C/C | C/C | C/C | C/C | C/P |
| S242 | S | C/P | C/P | C/P | C/P | C/C | C/C | C/C | C/C |
| S285 | S | C/P | C/P | C/P | C/P | C/C | C/C | C/C | C/C |
