# Supplementary material for: The broad-spectrum rice blast resistance (R) gene Pita2 encodes a novel R protein unique from Pita
Source: Rice (N Y). 2020 Mar 13;13:19. doi: 10.1186/s12284-020-00377-5 (PMC7070119; doi:10.1186/s12284-020-00377-5)
Supplement: Supplementary file 2 — Additional file 2: Table S2. Genotype and phenotype of 6 recombinants in the second round of genetic analysis [file 12284_2020_377_MOESM2_ESM.docx]

| **Table S2** Genotype and phenotype of 6 recombinants in the second round of genetic analysis. ^a^The phenotype of F_4_ descendant population derived from F_3_ segregating progeny were assessed to determine the recombinants at different markers. R/S: segregation in resistance (R) and susceptibility (S). C: CO39 pattern. P: IRBLta2-Re[CO] pattern. The location of *Pita* is between 12g 18150 and 12g18450. | | | | | | | | | |
| --- | --- | --- | --- | --- | --- | --- | --- | --- | --- |
| Recombinants | Phenotype^a^ | Genotype pattern resolved by different markers | | | | | | | |
|  |  | 12g18120 | 12g18150 | 12g18450 | 12g18530 | 12g18650 | 12g18920 | 12g19260 | 12g19304 |
| 4-3H9 | R | C/P | C/P | C/P | C/P | P/P | P/P | P/P | P/P |
| 5-2G10 | R | C/P | C/P | C/P | C/P | P/P | P/P | P/P | P/P |
| 6-4B6 | R | C/P | C/P | C/P | C/P | P/P | P/P | P/P | P/P |
| 7-3C1 | R/S | C/C | C/C | C/P | C/P | C/P | C/P | C/P | C/P |
| 8-2B3 | R/S | C/C | C/C | C/C | C/C | C/P | C/P | C/P | C/P |
| 8-2C8 | R/S | C/P | C/P | C/P | C/P | C/P | P/P | P/P | P/P |
