# Supplementary material for: The broad-spectrum rice blast resistance (R) gene Pita2 encodes a novel R protein unique from Pita
Source: Rice (N Y). 2020 Mar 13;13:19. doi: 10.1186/s12284-020-00377-5 (PMC7070119; doi:10.1186/s12284-020-00377-5)
Supplement: Supplementary file 3 — Additional file 3: Table S3. Candidate genes of Pita2 [file 12284_2020_377_MOESM3_ESM.docx]

| **Table S3** Candidate genes of *Pita2.* ^a^The predicted protein is smaller than 100 amino acids in length, hence not considered for further analysis. ^b^The predicted protein belongs to typical repetitive sequence that is not considered for further analysis. NA: not applicable. | | | | | | |
| --- | --- | --- | --- | --- | --- | --- |
| Gene model in IR64 | Feature of predicted genes | Gene model in Nipponbare | Sequence polymorphism compared to alleles in susceptible varieties | | | Candidate of *Pita2* |
|  |  |  | Nipponbare | CO39 | Unique polymer-phisms |  |
| IR64-1 | Unique | LOC_Os12g18530 | Yes | Yes | Yes | Flanking marker (excluded) |
| IR64-2 | Protein in small size ^a^ | NA | NA | NA | NA | Excluded |
| IR64-3 | Repetitive sequence^b^ | NA | NA | NA | NA | Excluded |
| IR64-4 | Unique | LOC_Os12g18560 | Yes | Yes | No | Excluded |
| IR64-5 | Repetitive sequence^b^ | NA | NA | NA | NA | Excluded |
| IR64-6 | Unique | NA | Yes | Yes | Yes | Candidate |
| IR64-7 | Unique | NA | Yes | Yes | Yes | Candidate |
| IR64-8 | Unique | NA | Yes | Yes | Yes | Candidate |
| IR64-9 | Unique | LOC_Os12g18630 | Yes | Yes | No | Excluded |
| IR64-10 | Unique | LOC_Os12g18640 | Yes | No | NA | Excluded |
| IR64-11 | Unique | LOC_Os12g18650 | Yes | No | NA | Excluded |
| IR64-12 | Protein in small size ^a^ | NA | NA | NA | NA | Excluded |
| IR64-13 | Repetitive sequence ^b^ | NA | NA | NA | NA | Excluded |
| IR64-14 | Protein in small size ^a^ | NA | NA | NA | NA | Excluded |
| IR64-15 | Unique | LOC_Os12g18690 | Yes | Yes | Yes | Candidate |
| IR64-16 | Unique | NA | Yes | Yes | Yes | Candidate |
| IR64-17 | Unique | LOC_Os12g18710 | Yes | Yes | No | Excluded |
| IR64-18 | Unique | LOC_Os12g18729 | Yes | Yes | Yes | Candidate |
| IR64-19 | Repetitive sequence ^b^ | NA | NA | NA | NA | Excluded |
| IR64-20 | Unique | LOC_Os12g18760 | Yes | Yes | Yes | Candidate |
| IR64-21 | Repetitive sequence ^b^ | NA | NA | NA | NA | Excluded |
| IR64-22 | Unique | LOC_Os12g18770 | Yes | Yes | Yes | Candidate |
| IR64-23 | Unique | LOC_Os12g18790 | Yes | Yes | Yes | Candidate |
| IR64-24 | Repetitive sequence ^b^ | NA | NA | NA | NA | Excluded |
| IR64-25 | Repetitive sequence ^b^ | NA | NA | NA | NA | Excluded |
| IR64-26 | Unique | LOC_Os12g18810 | Yes | No | NA | Excluded |
| IR64-27 | Repetitive sequence ^b^ | NA | NA | NA | NA | Excluded |
| IR64-28 | Unique | LOC_Os12g18820 | No | NA | NA | Excluded |
| IR64-29 | Repetitive sequence ^b^ | NA | NA | NA | NA | Excluded |
| IR64-30 | Repetitive sequence ^b^ | NA | NA | NA | NA | Excluded |
| IR64-31 | Repetitive sequence ^b^ | NA | NA | NA | NA | Excluded |
| IR64-32 | Repetitive sequence ^b^ | NA | NA | NA | NA | Excluded |
| IR64-33 | Repetitive sequence ^b^ | NA | NA | NA | NA | Excluded |
| IR64-34 | Unique | LOC_Os12g18860 | No | NA | NA | Excluded |
| IR64-35 | Protein in small size ^a^ | NA | NA | NA | NA | Excluded |
| IR64-36 | Unique | LOC_Os12g18880 | No | NA | NA | Excluded |
| IR64-37 | Protein in small size ^a^ | NA | NA | NA | NA | Excluded |
| IR64-38 | Unique | LOC_Os12g18900 | No | NA | NA | Excluded |
| IR64-39 | Repetitive sequence ^b^ | NA | NA | NA | NA | Excluded |
| IR64-40 | Repetitive sequence ^b^ | NA | NA | NA | NA | Excluded |
| IR64-41 | Repetitive sequence ^b^ | NA | NA | NA | NA | Excluded |
| IR64-42 | Repetitive sequence ^b^ | NA | NA | NA | NA | Excluded |
| IR64-43 | Unique | LOC_Os12g18920 | Yes | Yes | Yes | Flanking marker (excluded) |
