# Supplementary material for: The broad-spectrum rice blast resistance (R) gene Pita2 encodes a novel R protein unique from Pita
Source: Rice (N Y). 2020 Mar 13;13:19. doi: 10.1186/s12284-020-00377-5 (PMC7070119; doi:10.1186/s12284-020-00377-5)
Supplement: Supplementary file 4 — Additional file 4: Fig. S1. Expression pattern of Pita2 haplotypes in different rice varieties. [file 12284_2020_377_MOESM4_ESM.pptx]

## Slide 1
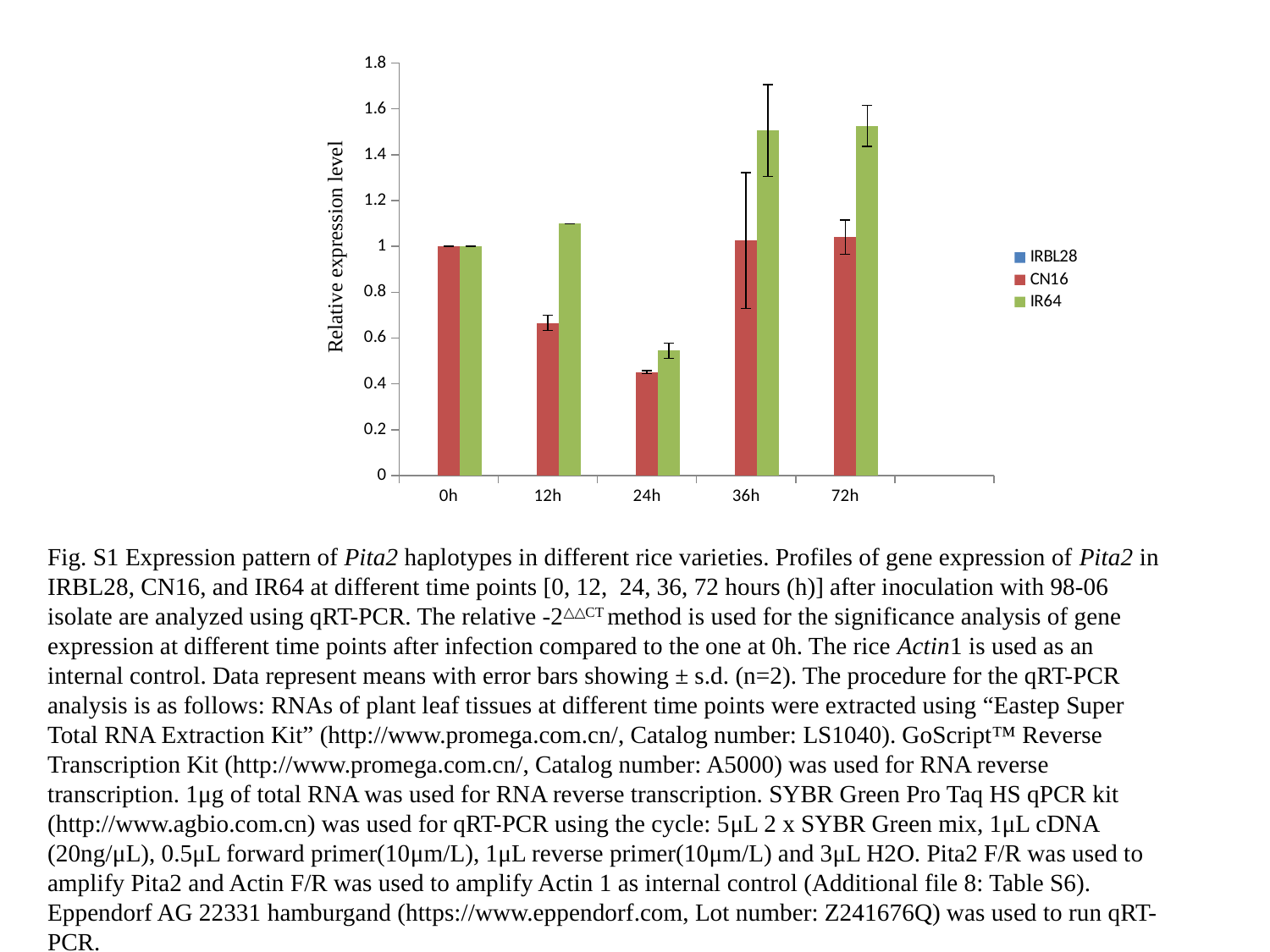

### Chart
| Category | IRBL28 | CN16 | IR64 |
|---|---|---|---|
| 0h | 1.0 | 1.0 | 1.0 |
| 12h | 0.8737239828588583 | 0.6659308245391604 | 1.0993595733252337 |
| 24h | 0.6078677385607495 | 0.4511969772053287 | 0.5447182009668626 |
| 36h | 1.3245987123057872 | 1.0260344740058593 | 1.5048079129645024 |
| 72h | 1.5023300417162033 | 1.0400292069773145 | 1.5257115679704074 |Relative expression level
Fig. S1 Expression pattern of Pita2 haplotypes in different rice varieties. Profiles of gene expression of Pita2 in IRBL28, CN16, and IR64 at different time points [0, 12, 24, 36, 72 hours (h)] after inoculation with 98-06 isolate are analyzed using qRT-PCR. The relative -2△△CT method is used for the significance analysis of gene expression at different time points after infection compared to the one at 0h. The rice Actin1 is used as an internal control. Data represent means with error bars showing ± s.d. (n=2). The procedure for the qRT-PCR analysis is as follows: RNAs of plant leaf tissues at different time points were extracted using “Eastep Super Total RNA Extraction Kit” (http://www.promega.com.cn/, Catalog number: LS1040). GoScript™ Reverse Transcription Kit (http://www.promega.com.cn/, Catalog number: A5000) was used for RNA reverse transcription. 1μg of total RNA was used for RNA reverse transcription. SYBR Green Pro Taq HS qPCR kit (http://www.agbio.com.cn) was used for qRT-PCR using the cycle: 5μL 2 x SYBR Green mix, 1μL cDNA (20ng/μL), 0.5μL forward primer(10μm/L), 1μL reverse primer(10μm/L) and 3μL H2O. Pita2 F/R was used to amplify Pita2 and Actin F/R was used to amplify Actin 1 as internal control (Additional file 8: Table S6). Eppendorf AG 22331 hamburgand (https://www.eppendorf.com, Lot number: Z241676Q) was used to run qRT-PCR.
