# Supplementary material for: The broad-spectrum rice blast resistance (R) gene Pita2 encodes a novel R protein unique from Pita
Source: Rice (N Y). 2020 Mar 13;13:19. doi: 10.1186/s12284-020-00377-5 (PMC7070119; doi:10.1186/s12284-020-00377-5)
Supplement: Supplementary file 5 — Additional file 5: Fig. S2. Phylogenetic tree analysis of the Pita2 haplotypes in different rice varieties. [file 12284_2020_377_MOESM5_ESM.pptx]

## Slide 1
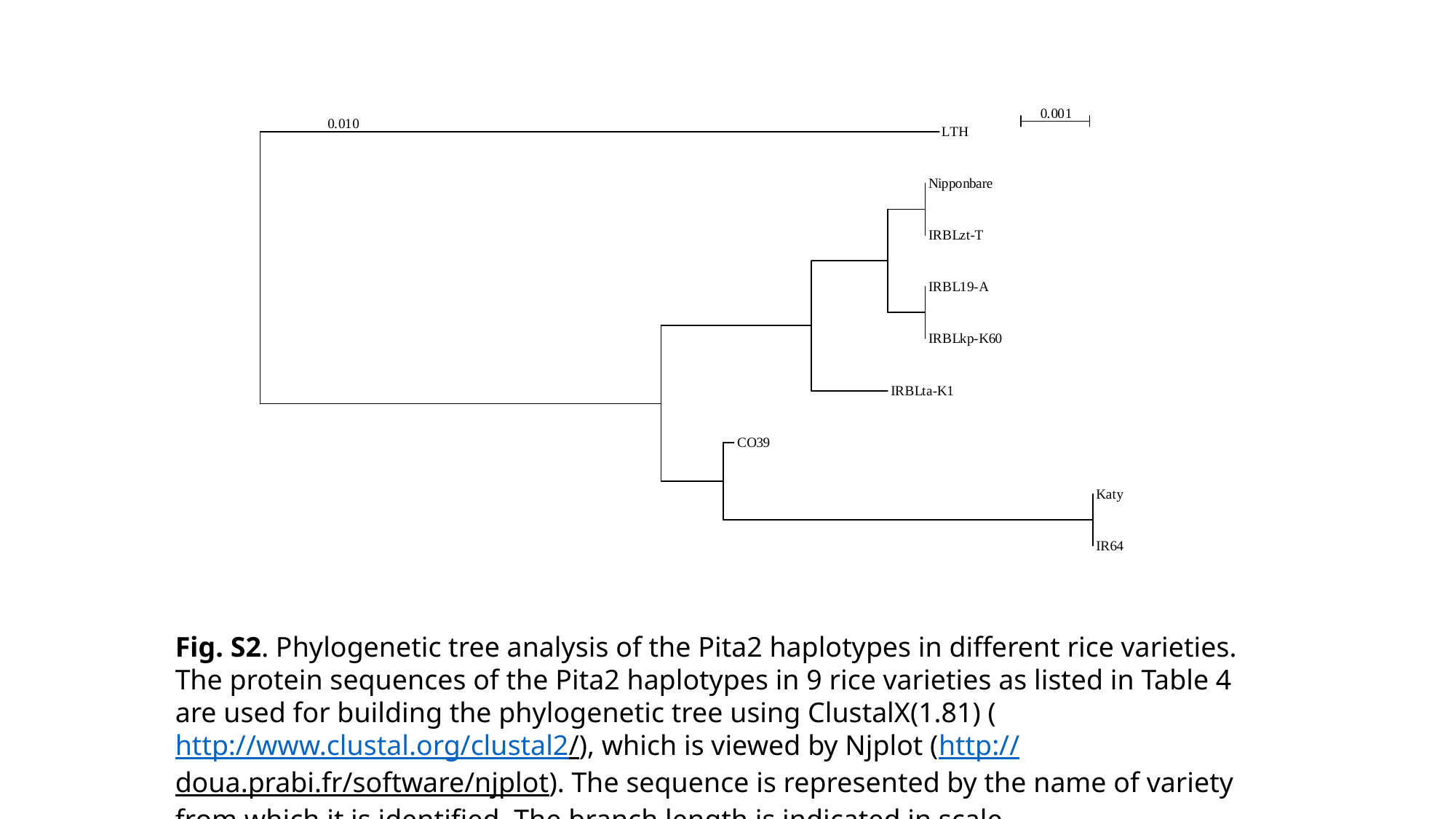

Fig. S2. Phylogenetic tree analysis of the Pita2 haplotypes in different rice varieties. The protein sequences of the Pita2 haplotypes in 9 rice varieties as listed in Table 4 are used for building the phylogenetic tree using ClustalX(1.81) (http://www.clustal.org/clustal2/), which is viewed by Njplot (http://doua.prabi.fr/software/njplot). The sequence is represented by the name of variety from which it is identified. The branch length is indicated in scale.
