# Supplementary material for: The broad-spectrum rice blast resistance (R) gene Pita2 encodes a novel R protein unique from Pita
Source: Rice (N Y). 2020 Mar 13;13:19. doi: 10.1186/s12284-020-00377-5 (PMC7070119; doi:10.1186/s12284-020-00377-5)
Supplement: Supplementary file 6 — Additional file 6: Fig. S3. Forty-six IRRI new released varieties were diagnosed by two Pita2 markers. [file 12284_2020_377_MOESM6_ESM.pptx]

## Slide 1
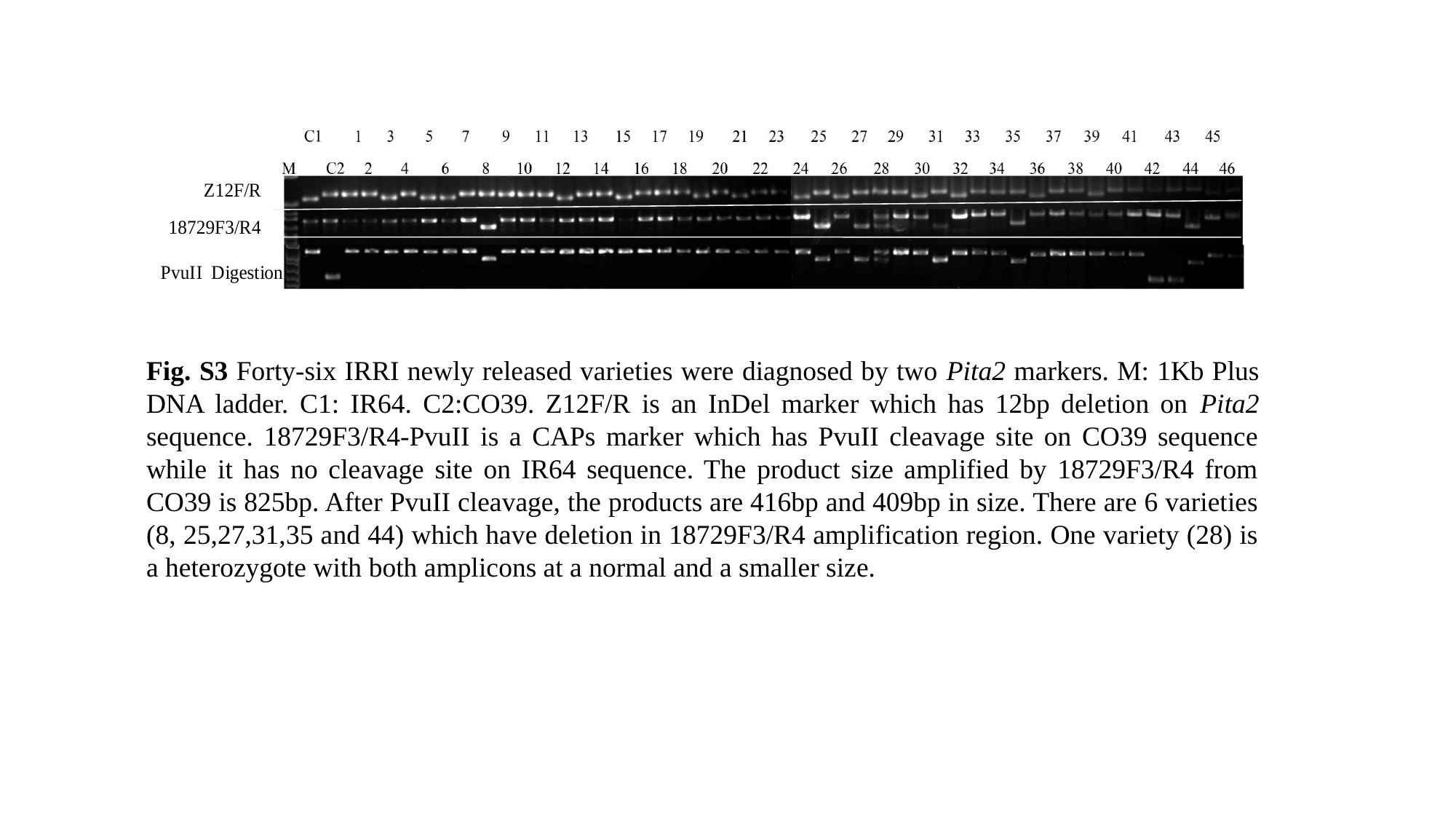

Fig. S3 Forty-six IRRI newly released varieties were diagnosed by two Pita2 markers. M: 1Kb Plus DNA ladder. C1: IR64. C2:CO39. Z12F/R is an InDel marker which has 12bp deletion on Pita2 sequence. 18729F3/R4-PvuII is a CAPs marker which has PvuII cleavage site on CO39 sequence while it has no cleavage site on IR64 sequence. The product size amplified by 18729F3/R4 from CO39 is 825bp. After PvuII cleavage, the products are 416bp and 409bp in size. There are 6 varieties (8, 25,27,31,35 and 44) which have deletion in 18729F3/R4 amplification region. One variety (28) is a heterozygote with both amplicons at a normal and a smaller size.
