# Supplementary material for: The broad-spectrum rice blast resistance (R) gene Pita2 encodes a novel R protein unique from Pita
Source: Rice (N Y). 2020 Mar 13;13:19. doi: 10.1186/s12284-020-00377-5 (PMC7070119; doi:10.1186/s12284-020-00377-5)
Supplement: Supplementary file 7 — Additional file 7: Table S4. Plant materials [file 12284_2020_377_MOESM7_ESM.docx]

| **Table S4** Plant materials used in this study. | | | | |
| --- | --- | --- | --- | --- |
| Plant materials | Target Gene | Donors | Recurrent Parents | Remark |
| IRBLa-A | *Pia* | AICHI ASAHI | LTH | *Pia* monogenic line |
| IRBLi-F5 | *Pii* | FUJISAKA 5 | LTH | *Pii* monogenic line |
| IRBLks-F5 | *Pik-s* | FUJISAKA 5 | LTH | *Piks* monogenic line |
| IRBLks-S | *Pik-s* | SHIN 2 | LTH | *Piks* monogenic line |
| IRBLk-ka | *Pik* | KANTO 51 | LTH | *Pik* monogenic line |
| IRBLkp-K60 | *Pik-p* | K 60 | LTH | *Pik-p* monogenic line |
| IRBLkh-K3 | *Pik-h* | K 3 | LTH | *Pik-h* monogenic line |
| IRBLz-Fu | *Piz* | FUKUNISHIKI | LTH | *Piz* monogenic line |
| IRBLz5-CA | *Piz5* | C101A51 | LTH | *Piz5* monogenic line |
| IRBLzt-T | *Piz-t* | TORIDE 1 | LTH | *Piz-t* monogenic line |
| IRBLta-CT2 | *Pita* | C105TTP2L9 | LTH | *Pita* monogenic line |
| IRBLb-B | *Pib* | BL 1 | LTH | *Pib* monogenic line |
| IRBLt-K59 | *Pit* | K 59 | LTH | *Pit* monogenci line |
| IRBLsh-S | *Pish* | SHIN 2 | LTH | *Pish* monogenic line |
| IRBL1-CL | *Pi1* | C101LAC | LTH | *Pi1* monogenic line |
| IRBL3-CP4 | *Pi3* | C104PKT | LTH | *Pi3* monogenic line |
| IRBL5-M | *Pi5*(t) | RIL 249 (Moro.) | LTH | *Pi5(t)* monogenic line |
| IRBL7-M | *Pi7*(t) | RIL 29 (Moro.) | LTH | *Pi7(t)* monogenic line |
| IRBL9-W | *Pi9* | WHD-1S-75-1-127 | LTH | *Pi9* monogenic line |
| IRBL19-A | *Pi19* | AICHI ASAHI | LTH | *Pi19* monogenic line |
| IRBLkm-Ts | *Pik-m* | TSUYUAKE | LTH | *Pik-m* monogenic line |
| IRBL20-IR24 | *Pi20* | ARL 24 | LTH | *Pi20* monogenic line |
| IRBLta2-Re | *Pita2* | REIHO | LTH | *Pita2* monogenic line |
| IRBL11-Zh | *Pi11*(t) | ZHAIYEQING | LTH | *Pi11(t)* monogenic line |
| IRBLta-Zh | *Pita* | Zhaiyeqing 8 | LTH | *Pita* monogenic line |
| IRBLta2-Re[CO] | *Pita2* | REIHO | CO39 | *Pita2* near isogenci line |
| IR64 |  |  |  | *Pita* and *Pita2* containing variety |
| #337 |  |  |  | IR64 EMS mutant |
| #6599 |  |  |  | IR64 EMS mutant |
| NSIC Rc 216 |  |  |  | IRRI newly released variety |
| NSIC Rc 222 |  |  |  | IRRI newly released variety |
| NSIC Rc 224 |  |  |  | IRRI newly released variety |
| NSIC Rc 226 |  |  |  | IRRI newly released variety |
| NSIC Rc 238 |  |  |  | IRRI newly released variety |
| NSIC Rc 240 |  |  |  | IRRI newly released variety |
| NSIC Rc 298 |  |  |  | IRRI newly released variety |
| NSIC Rc 300 |  |  |  | IRRI newly released variety |
| NSIC Rc 302 |  |  |  | IRRI newly released variety |
| NSIC Rc 308 |  |  |  | IRRI newly released variety |
| NSIC Rc 352 |  |  |  | IRRI newly released variety |
| NSIC Rc 356 |  |  |  | IRRI newly released variety |
| NSIC Rc 182 |  |  |  | IRRI newly released variety |
| NSIC Rc 184 |  |  |  | IRRI newly released variety |
| NSIC Rc 290 |  |  |  | IRRI newly released variety |
| NSIC Rc 294 |  |  |  | IRRI newly released variety |
| NSIC Rc 296 |  |  |  | IRRI newly released variety |
| NSIC Rc 324 |  |  |  | IRRI newly released variety |
| NSIC Rc 328 |  |  |  | IRRI newly released variety |
| NSIC Rc 330 |  |  |  | IRRI newly released variety |
| NSIC Rc 332 |  |  |  | IRRI newly released variety |
| NSIC Rc 334 |  |  |  | IRRI newly released variety |
| NSIC Rc 336 |  |  |  | IRRI newly released variety |
| NSIC Rc 338 |  |  |  | IRRI newly released variety |
| NSIC Rc 340 |  |  |  | IRRI newly released variety |
| NSIC Rc 392 |  |  |  | IRRI newly released variety |
| NSIC Rc 192 |  |  |  | IRRI newly released variety |
| NSIC Rc 272 |  |  |  | IRRI newly released variety |
| NSIC Rc 274 |  |  |  | IRRI newly released variety |
| NSIC Rc 278 |  |  |  | IRRI newly released variety |
| NSIC Rc 280 |  |  |  | IRRI newly released variety |
| NSIC Rc 282 |  |  |  | IRRI newly released variety |
| NSIC Rc 284 |  |  |  | IRRI newly released variety |
| NSIC Rc 286 |  |  |  | IRRI newly released variety |
| NSIC Rc 288 |  |  |  | IRRI newly released variety |
| NSIC Rc 238 |  |  |  | IRRI newly released variety |
| NSIC Rc 346 |  |  |  | IRRI newly released variety |
| NSIC Rc 25 |  |  |  | IRRI newly released variety |
| PSB Rc 18-Sub1 |  |  |  | IRRI newly released variety |
| NSIC Rc 21SR |  |  |  | IRRI newly released variety |
| NSIC Rc 218SR |  |  |  | IRRI newly released variety |
| NSIC Rc 220SR |  |  |  | IRRI newly released variety |
| NSIC Rc 304SR |  |  |  | IRRI newly released variety |
| NSIC Rc 342SR |  |  |  | IRRI newly released variety |
| NSIC Rc 344SR |  |  |  | IRRI newly released variety |
| Mestiso 30 |  |  |  | IRRI newly released variety |
