# Supplementary material for: The broad-spectrum rice blast resistance (R) gene Pita2 encodes a novel R protein unique from Pita
Source: Rice (N Y). 2020 Mar 13;13:19. doi: 10.1186/s12284-020-00377-5 (PMC7070119; doi:10.1186/s12284-020-00377-5)
Supplement: Supplementary file 8 — Additional file 8: Table S5. Isolates [file 12284_2020_377_MOESM8_ESM.docx]

| **Table S5** Isolates of *Magnaporthe oryzae* used in this study. | | |
| --- | --- | --- |
| Isolate ID | Collect year | Collected place |
|  |  |  |
| V86010 | 1986 | Camarines Sur |
| 9475-1-3 | 2014 | Ubay ,Bohol |
| BN111 | 1990 | Los Banos, Laguna |
| M64-1-3-9-1 | 1995 | Los Banos, Laguna |
| B90019 | 1990 | Caliraya, Laguna |
| B90187 | 1991 | Zamboanga del Sur |
| M39-1-3-8-1 | 1995 | Los Banos, Laguna |
| JMB8401 | 1985 | Caliraya, Lagun |
| JMB840610 | 1984 | Cuenca, Batangas |
| M39-1-2-21-2 | 1995 | Los Banos, Laguna |
| B90027 | 1990 | Caliraya, Laguna |
| 92319-9 | 2014 | Ubay ,Bohol |
| B90033 | 1990 | Caliraya, Laguna |
| IK81-3 | 1981 | Los Banos, Laguna |
| IK81-25 | 1981 | - |
| M101-1-2-9-1 | 1995 | Los Banos, Laguna |
| B90036 | 1990 | Caliraya, Laguna |
| B90002 | 1990 | Caliraya, Laguna |
| B90165 | 1991 | Blast Nursery |
| B90324 | 1991 | Blast Nursery |
| V850196 | 1985 | Caliraya, Laguna |
| C9240-4 | 1992 | Caliraya, Laguna |
| V850231 | 1985 | Caliraya, Laguna |
| 92520-7 | 1992 | Blast Nursery |
| CA89 | 1990 | Caliraya, Lagun |
| BN209 | 1990 | Los Banos, Laguna |
| PO6-6 | 1980 | Los Banos, Laguna |
| M015-1 | 2015 | Ubay ,Bohol |
| M015-2 | 2015 | Ubay ,Bohol |
| M015-3 | 2015 | Ubay ,Bohol |
| M015-4 | 2015 | Ubay ,Bohol |
| M015-5 | 2015 | Ubay ,Bohol |
| M015-6 | 2015 | Ubay ,Bohol |
| M015-7 | 2015 | Ubay ,Bohol |
| M015-8 | 2015 | Ubay ,Bohol |
| M015-9 | 2015 | Ubay ,Bohol |
| M015-10 | 2015 | Ubay ,Bohol |
| M015-11 | 2015 | Ubay ,Bohol |
| M015-12 | 2015 | Ubay ,Bohol |
| M015-13 | 2015 | Ubay ,Bohol |
| M015-14 | 2015 | Ubay ,Bohol |
| M015-15 | 2015 | Ubay ,Bohol |
| M015-16 | 2015 | Ubay ,Bohol |
| M015-17 | 2015 | Ubay ,Bohol |
| M015-19 | 2015 | Ubay ,Bohol |
| M015-20 | 2015 | Ubay ,Bohol |
| M015-21 | 2015 | Ubay ,Bohol |
| M015-22 | 2015 | Ubay ,Bohol |
| M015-23 | 2015 | Ubay ,Bohol |
| M015-24 | 2015 | Ubay ,Bohol |
| M015-25 | 2015 | Ubay ,Bohol |
| M015-26 | 2015 | Ubay ,Bohol |
| M015-27 | 2015 | Ubay ,Bohol |
| M015-28 | 2015 | Ubay ,Bohol |
| M015-29 | 2015 | Ubay ,Bohol |
| M015-30 | 2015 | Ubay ,Bohol |
| M015-31 | 2015 | Ubay ,Bohol |
| M015-32 | 2015 | Ubay ,Bohol |
| M015-33 | 2015 | Ubay ,Bohol |
| M015-34 | 2015 | Ubay ,Bohol |
| M015-35 | 2015 | Ubay ,Bohol |
| M015-36 | 2015 | Ubay ,Bohol |
| M015-37 | 2015 | Ubay ,Bohol |
| M015-38 | 2015 | Ubay ,Bohol |
| M015-39 | 2015 | Ubay ,Bohol |
| M015-40 | 2015 | Ubay ,Bohol |
| M015-41 | 2015 | Ubay ,Bohol |
| M015-42 | 2015 | Ubay ,Bohol |
| M015-43 | 2015 | Ubay ,Bohol |
| M015-44 | 2015 | Ubay ,Bohol |
| M015-45 | 2015 | Ubay ,Bohol |
| M015-46 | 2015 | Ubay ,Bohol |
| M015-47 | 2015 | Ubay ,Bohol |
| M015-48 | 2015 | Ubay ,Bohol |
| M015-49 | 2015 | Ubay ,Bohol |
| M015-50 | 2015 | Ubay ,Bohol |
| M015-51 | 2015 | Ubay ,Bohol |
| M015-52 | 2015 | Ubay ,Bohol |
| M015-53 | 2015 | Ubay ,Bohol |
| M015-54 | 2015 | Ubay ,Bohol |
| M015-55 | 2015 | Ubay ,Bohol |
| M015-56 | 2015 | Ubay ,Bohol |
| M015-57 | 2015 | Ubay ,Bohol |
| M015-58 | 2015 | Ubay ,Bohol |
| M015-59 | 2015 | Ubay ,Bohol |
| M015-60 | 2015 | Ubay ,Bohol |
| M015-61 | 2015 | Ubay ,Bohol |
| M015-62 | 2015 | Ubay ,Bohol |
| M015-63 | 2015 | Ubay ,Bohol |
| M015-64 | 2015 | Ubay ,Bohol |
| M015-65 | 2015 | Ubay ,Bohol |
| M015-66 | 2015 | Ubay ,Bohol |
| M015-67 | 2015 | Ubay ,Bohol |
| M015-68 | 2015 | Ubay ,Bohol |
| M015-69 | 2015 | Ubay ,Bohol |
| M015-70 | 2015 | Ubay ,Bohol |
| M015-71 | 2015 | Ubay ,Bohol |
| M015-72 | 2015 | Ubay ,Bohol |
| M015-73 | 2015 | Ubay ,Bohol |
| M015-74 | 2015 | Ubay ,Bohol |
| M015-75 | 2015 | Ubay ,Bohol |
| M015-76 | 2015 | Ubay ,Bohol |
| M015-77 | 2015 | Ubay ,Bohol |
| M015-78 | 2015 | Ubay ,Bohol |
| M015-79 | 2015 | Ubay ,Bohol |
| M015-80 | 2015 | Ubay ,Bohol |
| M015-81 | 2015 | Ubay ,Bohol |
| M015-82 | 2015 | Ubay ,Bohol |
| M015-83 | 2015 | Ubay ,Bohol |
| M015-84 | 2015 | Ubay ,Bohol |
| M015-85 | 2015 | Ubay ,Bohol |
| M015-86 | 2015 | Ubay ,Bohol |
| M015-87 | 2015 | Ubay ,Bohol |
| M015-88 | 2015 | Ubay ,Bohol |
| M015-89 | 2015 | Ubay ,Bohol |
| M015-90 | 2015 | Ubay ,Bohol |
| M015-91 | 2015 | Ubay ,Bohol |
| M015-92 | 2015 | Ubay ,Bohol |
| M015-93 | 2015 | Ubay ,Bohol |
| M015-94 | 2015 | Ubay ,Bohol |
| M015-95 | 2015 | Ubay ,Bohol |
| M015-96 | 2015 | Ubay ,Bohol |
| M015-97 | 2015 | Ubay ,Bohol |
| M015-98 | 2015 | Ubay ,Bohol |
| M015-99 | 2015 | Ubay ,Bohol |
| M015-100 | 2015 | Ubay ,Bohol |
| M015-101 | 2015 | Ubay ,Bohol |
| M015-102 | 2015 | Ubay ,Bohol |
| M015-103 | 2015 | Ubay ,Bohol |
| M015-104 | 2015 | Ubay ,Bohol |
| M015-105 | 2015 | Ubay ,Bohol |
| M015-106 | 2015 | Ubay ,Bohol |
| M015-107 | 2015 | Ubay ,Bohol |
| M015-108 | 2015 | Ubay ,Bohol |
| M015-109 | 2015 | Ubay ,Bohol |
| M015-110 | 2015 | Ubay ,Bohol |
| M015-111 | 2015 | Ubay ,Bohol |
| M015-112 | 2015 | Ubay ,Bohol |
| M015-113 | 2015 | Ubay ,Bohol |
| M015-114 | 2015 | Ubay ,Bohol |
| M015-115 | 2015 | Ubay ,Bohol |
| M015-116 | 2015 | Ubay ,Bohol |
| M015-117 | 2015 | Ubay ,Bohol |
| M015-118 | 2015 | Ubay ,Bohol |
| M015-119 | 2015 | Ubay ,Bohol |
| M015-120 | 2015 | Ubay ,Bohol |
| M015-121 | 2015 | Ubay ,Bohol |
| M015-122 | 2015 | Ubay ,Bohol |
| M015-123 | 2015 | Ubay ,Bohol |
| M015-124 | 2015 | Ubay ,Bohol |
| M015-125 | 2015 | Ubay ,Bohol |
| M015-126 | 2015 | Ubay ,Bohol |
| M015-127 | 2015 | Ubay ,Bohol |
| M015-128 | 2015 | Ubay ,Bohol |
| M015-129 | 2015 | Ubay ,Bohol |
| M015-130 | 2015 | Ubay ,Bohol |
| M015-131 | 2015 | Ubay ,Bohol |
| M015-132 | 2015 | Ubay ,Bohol |
| M015-133 | 2015 | Ubay ,Bohol |
| M015-134 | 2015 | Ubay ,Bohol |
| M015-135 | 2015 | Ubay ,Bohol |
| M015-136 | 2015 | Ubay ,Bohol |
| M015-137 | 2015 | Ubay ,Bohol |
| M015-138 | 2015 | Ubay ,Bohol |
| M015-139 | 2015 | Ubay ,Bohol |
| M015-140 | 2015 | Ubay ,Bohol |
| M015-141 | 2015 | Ubay ,Bohol |
| M015-142 | 2015 | Ubay ,Bohol |
| M015-143 | 2015 | Ubay ,Bohol |
| M015-144 | 2015 | Ubay ,Bohol |
| M015-145 | 2015 | Ubay ,Bohol |
| M015-146 | 2015 | Ubay ,Bohol |
| M015-147 | 2015 | Ubay ,Bohol |
| M015-148 | 2015 | Ubay ,Bohol |
| M015-149 | 2015 | Ubay ,Bohol |
| M015-150 | 2015 | Ubay ,Bohol |
| M015-151 | 2015 | Ubay ,Bohol |
| M015-152 | 2015 | Ubay ,Bohol |
| M015-153 | 2015 | Ubay ,Bohol |
| M015-154 | 2015 | Ubay ,Bohol |
| M015-155 | 2015 | Ubay ,Bohol |
| M015-156 | 2015 | Ubay ,Bohol |
| M015-157 | 2015 | Ubay ,Bohol |
| M015-158 | 2015 | Ubay ,Bohol |
| M015-159 | 2015 | Ubay ,Bohol |
| M015-160 | 2015 | Ubay ,Bohol |
| M015-161 | 2015 | Ubay ,Bohol |
| M015-162 | 2015 | Ubay ,Bohol |
| M015-163 | 2015 | Ubay ,Bohol |
| M015-164 | 2015 | Ubay ,Bohol |
| M015-165 | 2015 | Ubay ,Bohol |
| M015-166 | 2015 | Ubay ,Bohol |
| M015-167 | 2015 | Ubay ,Bohol |
| M015-168 | 2015 | Ubay ,Bohol |
| M015-169 | 2015 | Ubay ,Bohol |
| M015-170 | 2015 | Ubay ,Bohol |
| M015-171 | 2015 | Ubay ,Bohol |
| M015-172 | 2015 | Ubay ,Bohol |
| M015-173 | 2015 | Ubay ,Bohol |
| M015-174 | 2015 | Ubay ,Bohol |
| M015-175 | 2015 | Ubay ,Bohol |
| M015-176 | 2015 | Ubay ,Bohol |
| M015-177 | 2015 | Ubay ,Bohol |
| M015-178 | 2015 | Ubay ,Bohol |
| M015-179 | 2015 | Ubay ,Bohol |
| M015-180 | 2015 | Ubay ,Bohol |
| M015-181 | 2015 | Ubay ,Bohol |
| M015-182 | 2015 | Ubay ,Bohol |
| M015-183 | 2015 | Ubay ,Bohol |
| M015-184 | 2015 | Ubay ,Bohol |
| M015-185 | 2015 | Ubay ,Bohol |
| M015-186 | 2015 | Ubay ,Bohol |
| M015-187 | 2015 | Ubay ,Bohol |
| M015-188 | 2015 | Ubay ,Bohol |
| M015-189 | 2015 | Ubay ,Bohol |
| M015-190 | 2015 | Ubay ,Bohol |
| M015-191 | 2015 | Ubay ,Bohol |
| M015-192 | 2015 | Ubay ,Bohol |
| M015-193 | 2015 | Ubay ,Bohol |
| M015-194 | 2015 | Ubay ,Bohol |
| M015-195 | 2015 | Ubay ,Bohol |
| M015-196 | 2015 | Ubay ,Bohol |
| M015-197 | 2015 | Ubay ,Bohol |
| M015-198 | 2015 | Ubay ,Bohol |
| M015-199 | 2015 | Ubay ,Bohol |
| M015-200 | 2015 | Ubay ,Bohol |
| M015-201 | 2015 | Ubay ,Bohol |
| M015-202 | 2015 | Ubay ,Bohol |
| M015-203 | 2015 | Ubay ,Bohol |
| M015-204 | 2015 | Ubay ,Bohol |
| M015-205 | 2015 | Ubay ,Bohol |
| M015-206 | 2015 | Ubay ,Bohol |
| M015-207 | 2015 | Ubay ,Bohol |
| M015-208 | 2015 | Ubay ,Bohol |
| M015-209 | 2015 | Ubay ,Bohol |
| M015-210 | 2015 | Ubay ,Bohol |
| M015-211 | 2015 | Ubay ,Bohol |
| M015-212 | 2015 | Ubay ,Bohol |
| M015-213 | 2015 | Ubay ,Bohol |
| M015-214 | 2015 | Ubay ,Bohol |
| M015-215 | 2015 | Ubay ,Bohol |
| M015-216 | 2015 | Ubay ,Bohol |
| M015-217 | 2015 | Ubay ,Bohol |
| M015-218 | 2015 | Ubay ,Bohol |
| M015-219 | 2015 | Ubay ,Bohol |
| M015-220 | 2015 | Ubay ,Bohol |
| M015-221 | 2015 | Ubay ,Bohol |
| M015-222 | 2015 | Ubay ,Bohol |
| M015-223 | 2015 | Ubay ,Bohol |
| M015-224 | 2015 | Ubay ,Bohol |
| M015-225 | 2015 | Ubay ,Bohol |
| M015-226 | 2015 | Ubay ,Bohol |
| M015-227 | 2015 | Ubay ,Bohol |
| M015-228 | 2015 | Ubay ,Bohol |
| M015-229 | 2015 | Ubay ,Bohol |
| M015-230 | 2015 | Ubay ,Bohol |
| M015-231 | 2015 | Ubay ,Bohol |
| M015-232 | 2015 | Ubay ,Bohol |
| M015-233 | 2015 | Ubay ,Bohol |
| M015-234 | 2015 | Ubay ,Bohol |
| M015-235 | 2015 | Ubay ,Bohol |
| M015-236 | 2015 | Ubay ,Bohol |
| M015-237 | 2015 | Ubay ,Bohol |
| M015-238 | 2015 | Ubay ,Bohol |
| M015-239 | 2015 | Ubay ,Bohol |
| M015-240 | 2015 | Ubay ,Bohol |
| M015-241 | 2015 | Ubay ,Bohol |
| M015-242 | 2015 | Ubay ,Bohol |
| M015-243 | 2015 | Ubay ,Bohol |
| M015-244 | 2015 | Ubay ,Bohol |
| M015-245 | 2015 | Ubay ,Bohol |
| M015-246 | 2015 | Ubay ,Bohol |
| M015-247 | 2015 | Ubay ,Bohol |
| M015-248 | 2015 | Ubay ,Bohol |
| M015-249 | 2015 | Ubay ,Bohol |
| M015-250 | 2015 | Ubay ,Bohol |
| Mo17-1 | 2017 | Ubay ,Bohol |
| Mo17-2 | 2017 | Ubay ,Bohol |
| Mo17-3 | 2017 | Ubay ,Bohol |
| Mo17-4 | 2017 | Ubay ,Bohol |
| Mo17-5 | 2017 | Ubay ,Bohol |
| Mo17-6 | 2017 | Ubay ,Bohol |
| Mo17-7 | 2017 | Ubay ,Bohol |
| Mo17-8 | 2017 | Ubay ,Bohol |
| Mo17-9 | 2017 | Ubay ,Bohol |
| Mo17-10 | 2017 | Ubay ,Bohol |
| Mo17-11 | 2017 | Ubay ,Bohol |
| Mo17-12 | 2017 | Ubay ,Bohol |
| Mo17-13 | 2017 | Ubay ,Bohol |
| Mo17-14 | 2017 | Ubay ,Bohol |
| Mo17-15 | 2017 | Ubay ,Bohol |
| Mo17-16 | 2017 | Ubay ,Bohol |
| Mo17-17 | 2017 | Ubay ,Bohol |
| Mo17-18 | 2017 | Ubay ,Bohol |
| Mo17-19 | 2017 | Ubay ,Bohol |
| Mo17-20 | 2017 | Ubay ,Bohol |
| Mo17-21 | 2017 | Ubay ,Bohol |
| Mo17-22 | 2017 | Ubay ,Bohol |
| Mo17-23 | 2017 | Ubay ,Bohol |
| Mo17-24 | 2017 | Ubay ,Bohol |
| Mo17-25 | 2017 | Ubay ,Bohol |
| Mo17-26 | 2017 | Ubay ,Bohol |
| Mo17-27 | 2017 | Ubay ,Bohol |
| Mo17-28 | 2017 | Ubay ,Bohol |
| Mo17-29 | 2017 | Ubay ,Bohol |
| Mo17-30 | 2017 | Ubay ,Bohol |
| Mo17-31 | 2017 | Ubay ,Bohol |
| Mo17-32 | 2017 | Ubay ,Bohol |
| Mo17-33 | 2017 | Ubay ,Bohol |
| Mo17-34 | 2017 | Ubay ,Bohol |
| Mo17-35 | 2017 | Ubay ,Bohol |
| Mo17-36 | 2017 | Ubay ,Bohol |
| Mo17-37 | 2017 | Ubay ,Bohol |
| Mo17-38 | 2017 | Ubay ,Bohol |
| Mo17-39 | 2017 | Ubay ,Bohol |
| Mo17-40 | 2017 | Ubay ,Bohol |
| Mo17-41 | 2017 | Ubay ,Bohol |
| Mo17-42 | 2017 | Ubay ,Bohol |
| Mo17-43 | 2017 | Ubay ,Bohol |
| Mo17-44 | 2017 | Ubay ,Bohol |
| Mo17-45 | 2017 | Ubay ,Bohol |
| Mo17-46 | 2017 | Ubay ,Bohol |
| Mo17-47 | 2017 | Ubay ,Bohol |
| Mo17-48 | 2017 | Ubay ,Bohol |
| Mo17-49 | 2017 | Ubay ,Bohol |
| Mo17-50 | 2017 | Ubay ,Bohol |
| Mo17-51 | 2017 | Ubay ,Bohol |
| Mo17-52 | 2017 | Ubay ,Bohol |
| Mo17-53 | 2017 | Ubay ,Bohol |
| Mo17-54 | 2017 | Ubay ,Bohol |
| Mo17-55 | 2017 | Ubay ,Bohol |
| Mo17-56 | 2017 | Ubay ,Bohol |
| Mo17-57 | 2017 | Ubay ,Bohol |
| Mo17-58 | 2017 | Ubay ,Bohol |
| Mo17-59 | 2017 | Ubay ,Bohol |
| Mo17-60 | 2017 | Ubay ,Bohol |
| Mo17-61 | 2017 | Ubay ,Bohol |
| Mo17-62 | 2017 | Ubay ,Bohol |
| Mo17-63 | 2017 | Ubay ,Bohol |
| Mo17-64 | 2017 | Ubay ,Bohol |
| Mo17-65 | 2017 | Ubay ,Bohol |
| Mo17-66 | 2017 | Ubay ,Bohol |
| Mo17-67 | 2017 | Ubay ,Bohol |
| Mo17-68 | 2017 | Ubay ,Bohol |
| Mo17-69 | 2017 | Ubay ,Bohol |
| Mo17-70 | 2017 | Ubay ,Bohol |
| Mo17-71 | 2017 | Ubay ,Bohol |
| Mo17-72 | 2017 | Ubay ,Bohol |
| Mo17-73 | 2017 | Ubay ,Bohol |
| Mo17-74 | 2017 | Ubay ,Bohol |
| Mo17-75 | 2017 | Ubay ,Bohol |
| Mo17-76 | 2017 | Ubay ,Bohol |
| Mo17-77 | 2017 | Ubay ,Bohol |
| Mo17-78 | 2017 | Ubay ,Bohol |
