# Supplementary material for: The broad-spectrum rice blast resistance (R) gene Pita2 encodes a novel R protein unique from Pita
Source: Rice (N Y). 2020 Mar 13;13:19. doi: 10.1186/s12284-020-00377-5 (PMC7070119; doi:10.1186/s12284-020-00377-5)
Supplement: Supplementary file 9 — Additional file 9: Table S6. Markers [file 12284_2020_377_MOESM9_ESM.docx]

**Table S6.** Primers used for markers and candidate genes amplification. ^a^The physical location in Nipponbare genome (http://signal.salk.edu/cgi-bin/RiceGE5). ^b^Simple sequence repeats. ^c^Single-nucleotide polymorphism. ^d^Cleaved amplified polymorphic sequences, cleaved by ClaI. ^e^Insertion-deletion polymorphisms. ^f^The primers were used to amplify coding sequence of candidate genes. -: the sequence was absent in Nipponbare genome.

| Name | Sequence | Physical location^a^ | Remark |
| --- | --- | --- | --- |
| RM27920F | AAAGCGAGAAATCCGGAGATGG | 10139067-10139088 | SSR^b^ |
| RM27920R | TCCTCTCTCAAATCTCCTCGAAGC | 10139310-10139333 |  |
| RM1337F | GTGCAATGCTGAGGAGTATC | 11933312-11933331 | SSR |
| RM1337R | CTGAGAATCTGGAGTGCTTG | 11933503-11933522 |  |
| 12g17900F1 | ATGCTCGAACACTTTGCTAG | 10280812-10280831 | SNP^c^ |
| 12g17900R1 | CTTCCCTGTTCCCACTTTTC | 10280264-10280283 |  |
| 12g18110F2 | TACCCCTATTGTTAACTGTAG | 10417445-10417465 | SNP |
| 12g18110F2 | CATCTCACCCTATCTCTTGG | 10416470-10416489 |  |
| 12g18120F1 | GTCTACTTTTTTATTTCTATCTG | 10442410-10442432 | SNP |
| 12g18120R1 | GAGGGTGTGTTCGTAAATAG | 10441593-10441612 |  |
| 12g19304F2 | GCATTGAGCCACATTATCTAG | 11198566-11198586 | SNP/CAPS-ClaI^d^ |
| 12g19304R2 | TGGAATTGTGGAACTAAGATAC | 11197961-11197982 |  |
| 12g19590F1 | TCACATACTTAGTTCTGTTAC | 11419401-11419421 | SNP |
| 12g19590R1 | CAATTATCAGCAGAACAGAGAC | 11419827-11419848 |  |
| 12g20150F1 | AGTCATAATTGTTAGGTGAATC | 11733326-11733347 | SNP |
| 12g20150R1 | ATTATTTGGAAAAACAGACTGC | 11732573-11732594 |  |
| 12g18120 F3 | ACAGCCCTACACACGTTTTG | 10442196-10442215 | CAPS-NheI |
| 12g18120 R4 | ATGTAAAATGGGACGTGACG | 10441634-10441653 |  |
| 12g18150F | TCAGAGATTCAGAGCAGAG | 10469607-10469625 | SNP |
| 12g18150R | CCTGAGCATGGTCAAAAC | 10468211-10468228 |  |
| 12g18450F | ATGAAGTCACTTATTGGAAC | 10668149-10668168 | SNP |
| 12g18450R | CTATGTCAATCTCTCAAC | 10668731-10668748 |  |
| 12g18530F | TGTTCACAGTATACTCGAG | 10713834-10713852 | SNP |
| 12g18530R | ATCTTTCTCCTCTTCCATG | 10712206-10712224 |  |
| 12g18650F | TTTATAATGGCAGGTGAAG | 10770190-10770208 | SNP |
| 12g18650R | GATTCACCACATCCAAAC | 10771615-10771632 |  |
| 12g18920F | ATACACCGACAAGCTTTCG | 10979155-10979173 | SNP |
| 12g18920R | GTGTGGTTCAGTGGATGTG | 10977689-10977707 |  |
| 12g19260F | AGGCTGTTCGACGAGATG | 11166464-11166481 | SNP |
| 12g19260R | GCATTTGCTGGAATAAAACC | 11165530-11165549 |  |
| Z12F | TGCAGATTTGACTGCTCGGT | 10830614-10830633 | Indel^e^ |
| Z12R | GGGATCTTCCTCGCCCAAA | 10830821-10830839 |  |
| IR64-6F1 | GAGATGATCCATGAAGAGAATAG | - | IR64-6 amplification^f^ |
| IR64-6R1 | AGATGCGAAGAGGAACATATC | - |  |
| IR64-7F1 | GGTGAGGACGAGCTTCAGAC | 10735314-10735332 | IR64-7 amplification |
| IR64-7R1 | AAATCGGCTTCTGTCCTCCT | - |  |
| IR64-8F1 | TTACAACTGCAGTTTTATGATC | 10738078-10738099 | IR64-8 amplification |
| IR64-8R1 | CCCTTGAAAAAAAGCTCTCTTG | - |  |
| IR64-8F3 | GACATGCACGAAACGAGGAG | - |  |
| IR64-8R3 | GAAAAGGTATTACATGGAGC | 10741071-10741090 |  |
| IR64-15F1 | TATGACCTAAATACTAAAATCC | 10795766-10795787 | IR64-15 amplification |
| IR64-15R1 | CCTGTTTTTATCTTAGTTTGG | 10797652-10797672 |  |
| IR64-15 F3 | ATTCTATATCTCTACAATTTTAG | 10797716-10797738 |  |
| IR64-15 R3 | CAATTTACTTGGAGATGAAGC | 10799628-10799648 |  |
| IR64-15F5 | CAGGATCCGTTGGCTAGTC | 10796758-10796776 |  |
| IR64-15R6 | GGAAAAGTATGCAAAATAGAC | 10796287-10796307 |  |
| IR64-16F2 | CACGATCTCACCACCACACG | 10804395-10804414 | IR64-16 amplification |
| IR64-16R2 | CAAAAAATAATTAATCTTACCAG | 10806499-10806521 |  |
| IR64-20F1 | TCATTTTATCAATTACTTAACACC | 10845330-10845353 | IR64-20 amplification |
| IR64-20R1 | TGCATGCAAAGTAAGAACCATC | 10846376-10846397 |  |
| IR64-20F3 | CACCATGATAGTTTACTTGAAG | 10846670-10846691 |  |
| IR64-20R3 | AAATCATGTGCACATATTCTAC | 10848220-10848241 |  |
| IR64-22F1 | TGCCGTGTGTTTACCAGTAC | - | IR64-22 amplification |
| IR64-22R1 | ATCACGTCTTGCTTCTGTTGG | 10861698-10861718 |  |
| IR64-22F3 | GCATATGAGGAAGCTTCTC | 10862524-10862542 |  |
| IR64-22R3 | GATGTGTCAATGTTAGCATGG | 10864585-10864605 |  |
| IR64-22F5 | TAACAAATTGTGGAATAATCTTC | 10867349-10867371 |  |
| IR64-22R5 | CCCTCATTACTCCGCCAC | 10869575-10869592 |  |
| IR64-23F1 | TCATTGAGTTACAAAATTGTCC | 10871613-10871634 | IR64-23 amplification |
| IR64-23R1 | CCCTCTATCTCTTCCCCTTG | 10874743-10874762 |  |
| IR64-23F3 | CTAGCTATAGCAATGACATG | 10875824-10875843 |  |
| IR64-23R3 | TACCTCATTCTCCCCACCTC | - |  |
| 12g18729F1 | CTCTCTCAGATCTGGTGCGC | 10820160-10820179 | IR64-18 amplification |
| 12g18729R1 | GCGAAACTCCAAATGCTTGG | 10821760-10821779 |  |
| 12g18729F2 | GGTATCTGTCCTTTTAACCG | 10828120-10828139 |  |
| 12g18729R3 | ATCTGACAATGTAGAGGCTG | 10830219-10830238 |  |
| 12g18729F3 | ATCCATGCTTACTGAATATC | 10830352-10830371 | CAPS-PvuII |
| 12g18729R4 | TGTCAAACGTGGGTGTATGTG | 10831164-10831184 |  |
